# Sustainable supply chain partner selection and order allocation: A hybrid fuzzy PL-TODIM based MCGDM approach

**DOI:** 10.1371/journal.pone.0271194

**Published:** 2022-09-22

**Authors:** Shuqi Zhong, Jinxin Zhang, Xiaojun He, Sen Liu

**Affiliations:** 1 College of Innovative Business and Accountancy, Dhurakij Pundit University, Bangkok, Thailand; 2 School of Logistics, Yunnan University of Finance and Economics, Kunming, China; Universita degli Studi del Molise, ITALY

## Abstract

Sustainability, as a trend of social development and the embodiment of corporate social responsibility, has begun to receive more attention. To achieve this goal, sustainable supplier selection (SSS) and order allocation (OA) are seen as the crucial activities in corporate management. In the process of SSS, the psychological behavior of decision-makers (DMs) could play a critical role in the evaluation results. Therefore, introducing it into the decision-making process may lead to decision in line with the actual situation. In the uncertain multi-criteria group decision-making (MCGDM) problem described by probability linguistic term sets (PLTS), the DMs can evaluate the criteria of each supplier based on his own preference and hesitation, which is useful to avoid the loss of information. For this reason, this study develops a novel multi-criteria group decision-making combined with fuzzy multi-objective optimization (MCGDM-FMOO) model for SSS/OA problems by considering the triple bottom line (TBL) in which includes economic, environmental and social factors. The proposed method includes four stages. (1) the best-worst method (BWM) and entropy weight method are utilized to assign the weights of criteria to obtain the comprehensive weight. According to the output weights, the an acronym for interactive and multi-criteria decision-making in Portugese (TODIM) approach is applied to rank the suppliers under PLTS environment; (2) a FMOO model that can effectively deal with uncertainties and dynamic nature of parameter is formulated for allocating optimal order quantities; (3) two novel approaches are utilized to solve the FMOO model in order to obtain the richer Pareto frontier; and (4) the final OA solution is achieved by technique for order preference by similarity to ideal solution (TOPSIS) method. Finally, the validity and practicability of proposed MCGDM-FMOO model are verified by an example and comparative analysis with other classical MCGDM methods.

## 1 Introduction

To date, market competition has gradually risen from enterprise level to supply chain level, which drives companies to take supply chain management (SCM) measures to respond to the highly complex external environment [1]. SCM aims to plan, implement and control the supply chain network operations efficiently to deal with the recent rise in energy prices, industrial pollution and scarcity of raw material as well as the loss of natural resources, which implies the urgent need of sustainability supply chain management [2]. Therefore, sustainable supply chain management (SSCM) has been proposed as a novel concept and has also gradually attracted scholar’s attention [3, 4]. Generally speaking, SSCM is based on the needs of stakeholders to achieve economic, environmental and social sustainable development in three aspects of supply chains: logistics, cash flow and information flow [5].

With the segmentation of functions, there are a growing number of uneven suppliers in the market causing adverse impacts of disruption on enterprise operation [6]. Sustainable supplier selection (SSS) and order allocation (OA) are essential activities in SSCM that can significantly affect company efficiency and have an effect on profitability, flexibility, even agility [7]. Therefore, many leading companies have begun to consider sustainability when choosing suppliers. For example, as early as 2010, the retailer Wal-Mart required its supply chain to reduce carbon emissions, which challenged the suppliers to increase sustainability [8]. The cosmetics giant L’Oréal began to incorporate sustainability into its corporate strategy ten years ago; the company, which is committed to achieving zero-emission during production, packaging, transportation and sales. Fast fashion companies ZARA and H&M have always been synonymous with pollution and waste; in recent years, to fulfill their social responsibility to protect the environment, the two companies required suppliers to provide organic cotton as raw materials and established clothing recycling mechanism to reduce waste and pollution as much as possible in the clothing industry. In summary, to achieve better performance and higher competitiveness, SSS and OA needs to be taken into company’s management.

Supplier selection is the trigger in SSCM [9]. In the past few years, scholars have gradually shifted the most critical economic indicators affecting the supply chain to the perspective of environment and society, because only considering the economic factors of suppliers may lead to negative problems in a complex market environment. For example, Nike hired child labor in the 1990s which leads to negative impact on its goodwill. In the early 21st century, Foxconn workers committed suicide due to lack of labor rights protection. The addition of melamine to China’s Sanlu milk powder compromised the health of thousands of children. Schaeffer’s supply chain was interrupted due to excessive pollution from upstream suppliers which led to tremendous economic losses. According to above survey, economic, social and environmental factors may improve the performance of the supply chain. Therefore, this paper establishes an SSS criteria system considering all three dimensions of the TBL as a more comprehensive measure to improve sustainability. Economic criterion is usually based on the accounting factors of suppliers which reflects the current operating conditions. Environmental performance involves the efficiency of energy consumption, recycling and pollution control of various waste [8]. At the social level, one considers the impacts of business operations on human rights, labor habits, social organizations and residents [10].

To accurately express decision results of decision makers (DMs) when dealing with SSS, fuzzy logic is used as an effective tool in this article. However, DMs tend to utilize a single linguistic term (LT) to assess qualitative criterion; a common example is the Likert scale, which can limit the accuracy of an evaluation when facing the comprehensiveness of decision process. Hesitant fuzzy linguistic term sets (HFLTSs) can express DMs’ preferences multi-dimensionally which consists of positive and negative attitudes, but HFLTSs cannot clearly reflect the proportions or weights of different preferences for DMs. For this reason, Pang, Wang and Xu [11] put forward a probabilistic linguistic term set to help cope with this problem. PLTS requires the definition of multidimensional LTs and corresponding probabilities/weights to achieve improvement in accuracy of conveying the preference. In addition, PLTS can fully retain information of all DMs in group decision-making despite the group size.

Normally, a single supplier cannot meet all the procurement requirements of a company and maintain the stability of raw material supply. Therefore, companies need to purchase different types of products from multiple suppliers. This process is defined as multiple sourcing, which ensures the flexibility of the supply chain. During SSS, DMs often face a series of alternatives and conflicting criteria [12], including cost, service, etc. The above activity is regarded as MCGDM. In this research, the MCGDM approach is also adapted to formulate the SSS. Firstly, we apply a PLTS to the criteria weighting, which simultaneously describes the linguistic term set (LTS) of the evaluation information and the corresponding probability information. This effectively expresses fuzzy information in the real world [11]. Secondly, the subjective and objective weighting method on basis of the best-worst method (BWM) and the entropy weight method is developed to weight criteria. It is worth noting that the above weighting methods can also be used to assign weights of DMs. Finally, the TODIM method under the PLTS is applied to the SSS process to effectively deal with the uncertainties and risks in decision-making; consequently, the results will be closer to DMs’ preferences.

OA is the follow-up procedure of supplier selection, which belongs to typical multi-objective optimization practice. In the OA process, there are usually contradictions among the goals. For example, a decrease in cost usually means a decrease in service quality and an increased delivery time. In addition, in the real world, the objective is required to be as accurate as possible, and there are often uncertain criteria in the objective function. Fuzzy sets are a common solution used to deal with these uncertainties. Therefore, we propose a Multi-Objective Optimization (MOO) model that maximizes the purchasing value, minimizes the total cost, and minimizes carbon dioxide emissions. And the AUGMECON and LP-metrics methods are both utilized to find more solutions for conflicting uncertain multi-objective problems in the above mentioned.

Through the above research and analysis, this paper develops a new hybrid decision-making framework to deal with the SSS-OA problem. The research motivations are as follows:

(1) An important research object in MCGDM is the accurate criteria and expert weight. Considering that the SSS involves criteria in different fields, how to properly express the hesitation of DMs is an inevitable problem in weight distribution. In addition, the preferences of DMs and the data distribution show subjective and objective characteristics. Therefore, in technique selection, developing a weighting method that can integrate subjective preferences and objective characteristics is the main research motivation of this paper.

(2) The use of aggregation operator is a typical MCGDM problem. Most methods are based on the assumption that the DMs are absolutely rational, which is inappropriate and inconsistent with the actual situation. In real life, when people face the same probability of gains and losses, they will be more disgusted by losses, they also showed different attitude to risk. Therefore, it is necessary to develop a technical method that can not only consider the risk aversion behavior of DMs, but also show the hesitation degree of risk differentially.

(3) SSS and OA are coherent activities in the procurement process of enterprises. In practice, OA is usually determined based on the price provided by the supplier. However, considering the price can’t meet the current demand in the increasingly complex market environment. In addition, SSS and OA are usually regarded as two separate activities of different departments. But the mutual incoordination will lead to unreasonable order assignment results. The main challenge of this paper is how to take the SSS results into the OA process and build an assignment model that can deal with multiple objectives, so as to obtain more reasonable order allocation results.

In order to achieve the above motivations, the specific objectives of this paper are: (1) to improve the sustainability of the supply chain, this study constructs a criteria system based on TBL in the SSS selection. (2) to improve the accuracy of decision-making model, this study extends PLTS to MCGDM method, which can reflect the proportion of positive and negative attitudes of DMs effectively, so as to improve the accuracy of preference expression. (3) For the OA problem after SSS, we transform the multi supplier and multi product MOO into a fuzzy multi-objective optimization (FMOO) model to deal with the dynamic nature of parameters. Finally, the classic TOPSIS method is proposed to select the optimal solution from all the solution sets.

This paper proposes a framework from which we can derive insights into SSS-OA problems. Accordingly, this paper aims to contribute to the literature in four critical ways: (1) We propose a joint model of supplier selection and order allocation under the consideration of sustainability, which is applicable to conventional manufacturing industries such as household appliances, furniture production, electronic equipment and so on. According to the results of SSS, we construct the objective function of maximum purchase value, which effectively connects the problems of SSS and OA, and makes the two independent phases in SSCM become coherent decision-making activities. As a result, the joint model leads to the coordination between supplier selection stage and OA stage, and more reasonable assignment results can be obtained according to supplier ranking. (2) The PLTS can flexibly reflect the uncertainty and hesitation of DMs in information evaluation. The DMs involved in the MCGDM process focus on different fields, so they may lack reliable information about specific criteria, which makes it difficult for them to express their opinions in crisp number. In this case, PLTS can effectively help DMs convey their uncertain information with probability, so as to solve the fuzziness of qualitative evaluation and improve the accuracy. (3) The comprehensive weighting model combined with BWM and entropy method can not only be used for weighting a large number of criteria, but also give the weight distribution of all DMs. Both evaluation opinions and evaluation information of experts are considered in the weighting model, which can balance the subjective preference and objective contribution to the greatest extent, and improve the rationality and accuracy of the obtained weight. In addition, the above two techniques are based on PLTS, which can better reflect the uncertainty than the crisp number. (4) Based on prospect theory, an extended TODIM method considering DM behavior is proposed and applied in SSS. This technique takes the psychological behavior of DMs including reference dependence and loss aversion into account, that is, DMs tend to avoid losses rather than gain benefits. Accordingly, higher discrimination is reflected in the ranking results. It is more in line with the actual situation, so, it has certain theoretical significance and practical value.The specific arrangement of the study is as below. Chapter 2 reviews research direction and application of criteria and mathematical methods to SSS/OA problems in SSCM. Chapter 3 gives the explanation of fuzzy sets, PLTS and proposes the MCGDM-FMOO model. Chapter 4 applies the proposed MCGDM-FMOO model to an illustrative example. Chapter 5 discusses the outcomes and significance of this research. Chapter 6 summarizes our research and put forward next project.

## 2 Literature review

### 2.1 SSCM and SRM

SSCM attempts to enhance economic, environmental and social capability from the industry and value chain perspective, thereby effectively improving sustainability [10]. Stakeholders, government agencies and regulatory agencies are gradually becoming aware of the profound impact that the sustainability has on the environment and society [2]. Companies are also beginning to realize that supply chain sustainability will affect their image and reputation [5]. Therefore, the development of SSCM has become a demand of both individuals and society. Through a literature review, we found that SSCM is often associated with green supply chain management (GSCM). It is worth noting that SSCM and GSCM seem to be similar concepts, but their scope is different. GSCM mainly starts from the production process and carries out controls on pollution in each subsequent process, usually including procurement, involvement, production, packaging, image, etc. [12]. SSCM not only promotes the sustainability of a company’s production practices but also discusses its effects on production process with the three comprehensive aspects [13]. Therefore, we believe that SSCM research needs to be based on the TBL and that research needs to be raised from the corporate level to the social level.

Companies are increasingly dependent on suppliers, and the importance of supplier relationship management (SRM) has been highlighted due to globalization [14]. SRM is an important concept in SSCM. Like SSCM, SRM is also an academic term that means to manage the relationship between the participants in the supply chain to help participants jointly perform a series of operations, such as planning, operation, and decision-making, to improve performance and sustainability. SSS/OA is a critical research problem in SRM. However, many studies on SRM mainly focus on the relationship between SRM and corporate performance [1, 14, 15], the level of implementation of organizational supply chain management [16], the impact on carbon emissions, and the relationship with business operations [17]. TBL can help purchasers / managers evaluate suppliers across the economic, environmental and social perspectives., which may bring continuous improvement to the enterprise. So we attempt to construct a MCGDM-FMOO research framework to solve the SSS/OA problem based on the TBL theory.

### 2.2 Supplier selection criteria

To a large extent, the construction of the criteria system affects the validity and reliability of decision-making results, while incomplete criteria will have an adverse impact on DMs’ ability to accurately assess supplier sustainability [8]. Dickson and Gary [18] constructed the first relatively complete supplier selection criteria system containing 23 items and argued that cost, quality and delivery time belong to the critical considerations for evaluating suppliers. Weber [19] analyzed the most recent 74 papers and found conflicts among the selection criteria, concluding that supplier selection falls under the research area of multi criteria decision making. Chen [20] ranked the importance of 23 criteria based on previous studies and argued that quality, delivery and historical performance belong to the fundamental criteria. Therefore, based on this early research, delivery time, price and quality are considered to be the most indispensable factors in supplier selection process. Rashidi, Noorizadeh, Kannan and Cullinane [8] also proposed that quality, transportation, and price are considered the most critical criteria in the assessment of sustainability by performing a quantitative and qualitative analysis of related literature in the past 30 years.

However, many scholars have ignored the above traditional criteria in their research on SSS [21]. Govindan, Mina, Esmaeili and Gholami-Zanjani [22] emphasized quality and timely delivery in the supplier ranking but ignored cost. In some studies, the important role of supplier delivery capabilities has not been explored [23]. Not only economic performance needs to be considered, but environmental and social factors must not be ignored. Environmental management systems, recycling, pollution control, eco-design and energy consumption are the most critical and common criteria among the 45 commonly used environmental factors identified by Rashidi, Noorizadeh, Kannan and Cullinane [8]. Linton, Klassen and Jayaraman [24] linked the economy and environment together and pointed out that SSCM must be based on product whole life cycle, so total cost becomes a necessary consideration.

The negative impact of resources used and the pollutants generated also need to be considered in SSCM. Therefore, energy consumption and pollution control (especially carbon emissions) are evaluation factors that cannot be ignored when evaluating suppliers’ sustainability. Carbon emissions during transportation are another critical and easily overlooked factor [25]. In terms of society, the sustainability of suppliers is mainly reflected in three aspects: the influence of enterprise operations on employees, the influence on social organizations, and the influence on social members [8]. However, according to a literature review on SSS [26], many current studies have not fully considered and investigated social factors.

### 2.3 Approaches in the SSS/OA

Solving SSS/OA problems usually includes four methods [27]: MCGDM methods, mathematical programming(MP), artificial intelligence(AI) and hybrid methods. Among them, hybrid methods support DMs by using a combination of two or more methods of the same type [28, 29]. It also allows decision makers to integrate different types of methods to make up for the shortcomings of one approach, thereby improving the accuracy of decision-making [5, 6, 30–32].

Several representative studies on decision-making and order allocation problems are showed in Table 1. It can be seen that although more scholars began to focus on the tools of AI and MP, MCGDM method is still the mainstream and important technique for studying SSS. Accordingly, improving and combining the sub methods to make up for the defects is an important research trend at present.

**Table 1:** A summary of representative studies on supplier selection and order allocation.

	Authors	Application	Research objective	Technique
Single technique	Ghadimi et al. [33]	Medical supply chain	Make prompt decisions with less human interactions	AI
	Tozanli et al. [34]	Traditional supply chain	Proposes an Industry 4.0 setting for sustainable product recovery processes	MP
	Qin et al. [35]	Automobile supply chain	Construct an extended TODIM behavior decision method to green supplier selection	MCGDM
	Deshmukh and Sunnapwar [36]	Food supply chain	Revised FAHP is utilized to select best green supplier	MCGDM
Combined technique	Li et al. [37]	Water environment treatment	Propose a hybrid MCGDM model to select sustainable supplier	MCGDM
	Lo et al. [38]	Sustainable supply chain	Develops a two-stage MCGDM approach for sustainable supplier evaluation and transportation planning	MCGDM
	Pishchuloy et al. [39]	Sustainable supply chain	Integrate a revised AHP method and the comprehensive criteria system to evaluate performance of supplier	MCGDM
	Islam et al. [40]	Food supply chain	Conduct demand forecasting, SS-OA by ML	AI
	Kannan [9]	Textile supply chain	Explore the influence of multi stakeholders on the process of SSS	MCGDM
	Cheng et al. [41]	Traditional supply chain	Alleviate the workload on experts involved in supplier evaluation process by ML	MCGDM combined with AI
	Tong et al. [42]	Traditional supply chain	Construct a supplier selection evaluation framework for SMEs	MCGDM
	Hasan et al. [43]	Traditional supply chain	Develop a DSS that will help the DMs to select supplier and allocate order	MCGDM combined with MP

AI, Artificial Intelligence; MP, Mathematical Programming; TODIM, an acronym for interactive and multi-criteria decision-making in Portugese; MCGDM, Multi Criteria Group Decision-Making; DSS, Decision Support System; FAHP, Fuzzy analytic hierarchy process; AHP, analytic hierarchy process; ML, Machine Learning; SSS, sustainable supplier selection; SMEs, Small and medium-sized enterprises; DMs, decision makers.

MCGDM method integrates qualitative research methods and quantitative research methods, resulting in the balance between DMs’ subjective opinions and objective data. Therefore, it is widely used in multi industry for supplier selection and evaluation.

Through the research framework formed over the years, MCGDM is mainly discussed in three steps. Firstly, DMs select the appropriate language set to evaluate the criteria by scoring them based on the importance, so as to make the reasonable differences in weight distribution. DMs usually assess criteria with crisp number in the early stages of research. With the development of fuzzy set theory, experts can choose a language set more suitable for the scene to evaluation, which is an effective tool to solve the uncertainty and hesitation of DMs in this process. Secondly, another critical aspect in MCGDM is to extend language set into to weighting model. This procedure needs to take the DMs’ subjective preferences and the objective characteristics of data into account. Therefore, weighting model are usually divided into subjective and objective technique, which are also suitable for expert weighting. Finally, the aggregation operators are utilized for scoring and ranking each alternative. In general, MCGDM is the hybrid approach involving multiple management departments, then weighting DMs and criteria to gain a set of data for alternative evaluation.

MCGDM contains many subdivision methods, such as AHP, BWM, TOPSIS, DEA, TODIM, VIKOR etc. each single method has inevitable defects and irreplaceable advantages. Exploring the combination and improvement of different methods can realize the mutual complementarity, so as to gain more credible and reasonable criteria weighting and supplier ranking results. As an essential and concerned research field, MCGDM mainly focuses on processing of language set, weighting distribution and aggregation operator.TODIM, as a MCGDM method that can consider the psychological factors of DMs to avoid loss, is more in line with the application in real scenes. Qin, Liu and Pedrycz [35] expanded the TODIM method to deal with green supplier evaluation under IT2FS and performed sensitivity analysis on the results. Celik, Yucesan, & Gul [44] constructed the hybrid IT2FS-BWM-TODIM model to handle uncertainty in the decision-making of GSS, which is useful for textile industry stakeholders. Gomes, Machado, Santos and Caldeira [45] applied the original and extended TODIM approaches to supplier selection in the steel industry and proved that the two methods produced the same experimental results under certain circumstances. TODIM was extended to the unbalanced HFLTS language environment to solve the sorting problem of telecom service providers [46]. Feng and Gong [47] proposed a two-stage decision-making model that integrates the LEWM and MOP: the LEWM was used to ranking green supplier in the automobile manufacturing industry and to conduct order allocation from three aspects: total cost, carbon emission and purchase value. Dos Santos, Godoy and Campos [48] collected information on environmental standards proposed by 32 experts and applied the comprehensive MCGDM Entropy-TOPSIS-F approach to the furniture SSM. These methods chose green suppliers with the best environmental performance. Ecer and Pamucar [13] considered the important roles of the economy, society and environment in the sustainable supply chain and determined the weights of these three types of criteria by using F-BWM.

Through the integrated fuzzy CoCoSo method, electrical appliance manufacturers were ranked according to their pros and cons. From the perspective of the green innovation capabilities of SMEs, a framework for large organizations to select suppliers was constructed from three stages of criteria: construction, criteria weighting (BWM) and supplier ranking (fuzzy TOPSIS). Finally, correlation sensitivity analysis was obtained to prove the validity of the decision model [49].

By reviewing the literature, we found that the BWM, entropy and TODIM methods, three commonly employed MCGDM tools, have been widely used in the real world. However, the integrated BWM-Entropy model is used for weighting, and the decision model involving TODIM in the ranking process has not been developed. Therefore, we propose a hybrid MCGDM model to deal with the SSS process.

### 2.4 Research gaps and highlights

This literature shows that considerable studies have been performed on green supplier evaluation [50]; the works on SSS have demonstrated a rising trend of popularity in recent years, but the overall progress has been relatively small. In addition, in SSS-related research, social and environmental factors are often not given the same attention as economic performance. Meanwhile, some developing countries do not pay enough attention to SSCM due to lacking the formulation of relevant laws and regulations. Moreover, choosing a more sustainable supplier often means increased costs for companies.

Some single MCGDM approaches, such as AHP, BWM, Entropy, TODIM, TOPSIS are most commonly used to solve SSS problems. These methods need to be combined with other tools to solve problems more effectively [51]. However, many studies are based on a single approach under a fuzzy environment [2]. Furthermore, the prerequisites of bounded rationality for DMs were ignored in many MCGDM tools, while TODIM is an effective tool to take those factors into account.

Therefore, we construct the SSS/OA criteria based on the TBL and integrate the PL-BWM-Entropy-TODIM framework to promote SSS. Subsequently, an FMOO model for order allocation that can handle uncertain parameters is proposed. Then, two methods, AUGMECON and LP-metrics, are effective tools to gain a richer Pareto solution set. At last, we use the TOPSIS method to select the most appropriate Pareto solution.

Table 2 shows a comparison between this study and previous studies displaying the problems solved, the fuzzy environment, the sustainable criteria, and the MCGDM method used.

**Table 2:** Comparison of studies sustainable criteria and approaches.

Literature	Problem	Fuzzy	Sustainability			MCGDM		Integrated
			Eco	Env	Soc	Single	Hybrid	approaches
Lima Junior, Osiro [52]	SS	√	√			AHP		
Hamdan, Cheaitou [53]	GSS/OA	√	√	√		TOPSIS		Fuzzy TOPSIS+ MOILP
Orji, Wei [54]	SSS	√	√	√	√	TOPSIS		
Govindan, Sivakumar [55]	GSS/OA	√	√	√		TOPSIS		Fuzzy TOPSIS+ MOLP
Jauhar, Pant [56]	SSS		√	√		DEA		DEA+ Differential Evolution
Rao, Xiao [57]	SSS	√	√			VIKOR		Extended VIKOR
Banaeian, Mobli [58]	GSS	√	√	√		TOPSIS/VIKOR/GRA		
Vahidi, Torabi [23]	SSS/OA		√	√	√			Integrated SWOT-QFD
Song, Xu [59]	SSS	√	√	√	√	DEMATEL		Rough DEMATEL
This study	SSS/OA	√	√	√	√		BWM-Entropy+TODIM	PL- BWM-Entropy+PL-TODIM+FMOO

## 3 Proposed MCGDM-FMOO approach

**Fig 1:**
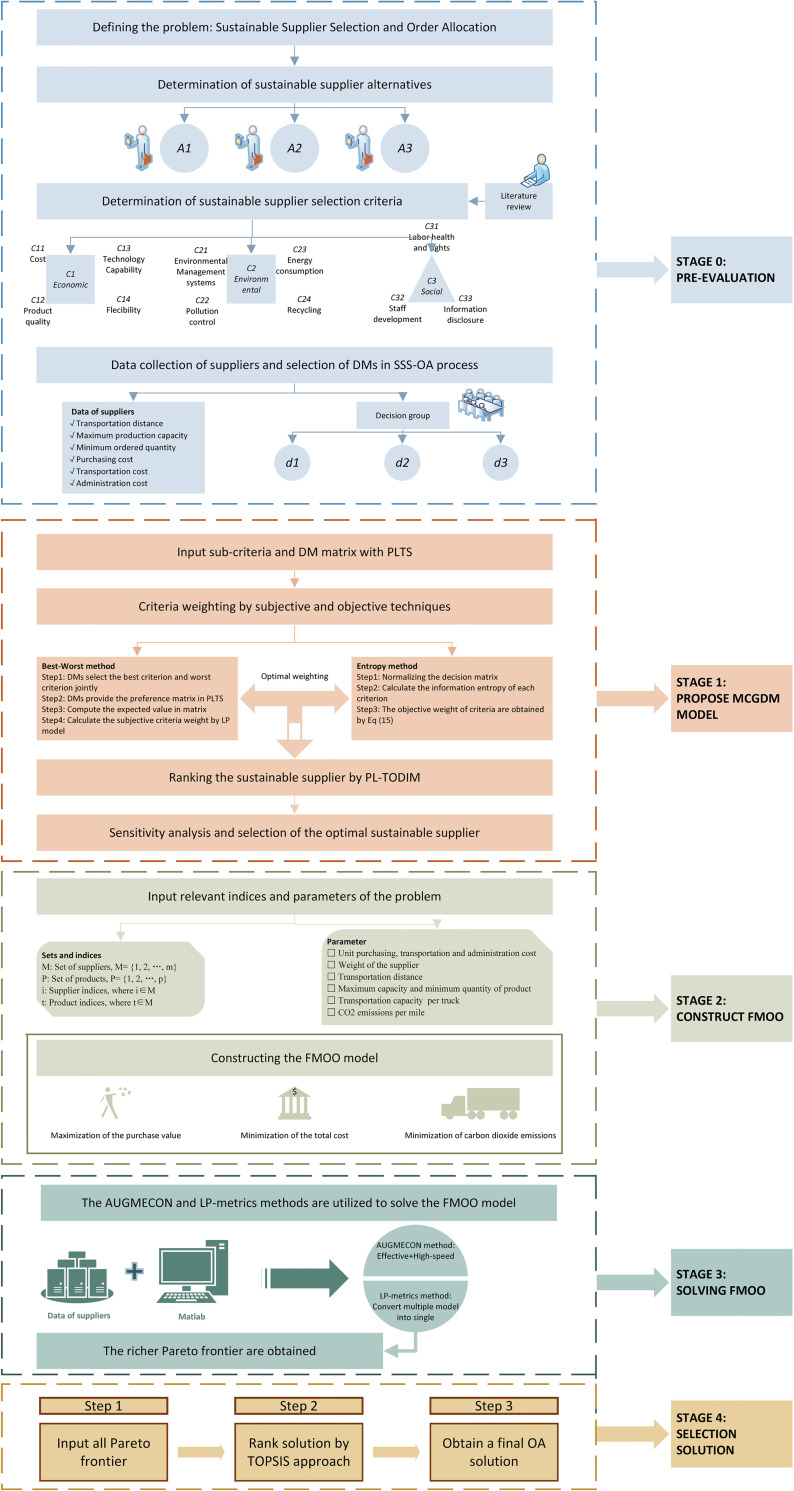
The process of MCGDM-FMOO approach.

The proposed approach includes four stages. The first stage integrates PL-BWM-Entropy and PL-TODIM into SSS. In the second stage, we propose the FMOO model to allocate the company’s purchase quantities among various suppliers. In the third stage, the AUGMECON and LP-metrics are utilized to solve FMOO model and obtain Pareto solutions. In the final stage, the optimal result from Pareto frontier is computed by TOPSIS. The MCGDM-FMOO framework in this study is shown in Fig 1.

### 3.1 Fuzzy logic

#### 3.1.1 Trapezoidal fuzzy number

**Definition 1 [60]:** Assuming $\tilde{g}$ is a fuzzy set in a universe of discourse *X*. $\mu_{\tilde{g}}(x)$ which denotes a membership function maps each element *x* to a real number in the interval [0,1].

**Definition 2 [60]:** Let $\tilde{g}=(g_{1},g_{2},g_{3},g_{4})$ a trapezoidal fuzzy number, the membership function $\mu_{\tilde{g}}(x)$ is given by:

$$\mu_{\tilde{g}}(x)=\{\begin{array}{l} \frac{x-g_{1}}{g_{2}-g_{1}}\;g_{1}<x<g_{2}\\ 1\;g_{2}<x<g_{3}\\ \frac{g_{4}-x}{g_{4}-a_{3}}\;g_{3}<x<g_{4}\\ 0\;otherwise \end{array}$$ (1)

**Definition 3:** The defuzzified value of $\tilde{g}=(g_{1},g_{2},g_{3},g_{4})$ is given by:

$$m(\tilde{a})=(\frac{g_{1}+g_{2}+g_{3}+g_{4}}{4})$$ (2)

### 3.1.2 Probabilistic linguistic term set (PLTS)

**Definition 4 [11].** Let $S=\{s_{l}|l=‐\tau ,\cdots ,‐1,0,1,\cdots ,\tau \}$ is a linguistic term set. *F*^(*l*)^*pr*^(*l*)^ is the LT *F*^(*l*)^ with probability *pr*^(*l*)^, #*F*(*pr*) represents quantity of LTs in *F*(*pr*). $F(pr)=\{F^{\left(l\right)}pr^{\left(l\right)}|F^{\left(l\right)}\in S,pr^{\left(l\right)}\geq 0,l=1,2,\cdots ,\#F(pr),\sum_{i=1}^{\#F(pr)}pr^{\left(l\right)}\leq 1\}$ denotes a PLTS.

**Definition 5 [11].** Let a PLTS $F(pr)=\{F^{\left(l\right)}pr^{\left(l\right)}|l=1,2,\cdots ,\#F(pr)\}$ with $\sum_{i=1}^{\#F(pr)}pr^{\left(l\right)}\leq 1$, *sub*^(*l*)^ denotes the subscript of LT *F*^(*l*)^. If $F^{\left(l\right)}pr^{\left(l\right)}(l=1,2,\cdots ,\#F(pr))$ are sorted through the values of *sub*^(*l*)^*pr*^(*l*)^ in descending order, we call *F*(*pr*) an ordered PLTS.

**Definition 6 [11].** Let a PLTS $F(pr)=\{F^{\left(l\right)}pr^{\left(l\right)}|l=1,2,\cdots ,\#F(pr)\}$ with $\sum_{i=1}^{\#F(pr)}pr^{\left(l\right)}\leq 1$. $\tilde{F}(\tilde{pr})=\{F^{\left(l\right)}{\tilde{pr}}^{\left(l\right)}|l=1,2,\cdots ,\#\tilde{F}(\tilde{pr})\}$ represents the standardized PLTS, where $\tilde{p}r^{\left(l\right)}=pr^{\left(l\right)}/\sum_{l=1}^{\#F(p)}pr^{\left(l\right)}$ for all l=1,2,⋯,#F(pr).

**Definition 7 [11].** Let $F_{1}(pr_{1})=\{F_{1}^{\left(l\right)}pr_{1}^{\left(l\right)}|l=1,2,\cdots ,\#F_{1}(pr_{1})\}$ and $F_{2}(pr_{2})=\{F_{2}^{\left(l\right)}pr_{2}^{\left(l\right)}|l=1,2,\cdots ,\#F_{2}(pr_{2})\}$ be any two PLTSs. If #*F*_1_(*pr*_1_)>#*F*_2_(*pr*_2_), #*F*_1_(*pr*_1_)−#*F*_2_(*pr*_2_) LTs are added to #*F*_2_(*pr*_2_) until the numbers of LTs in *F*_1_(*pr*_1_) and *F*_2_(*pr*_2_) are equal.

**Definition 8 [11].** Let a PLTS $F(pr)=\{F^{\left(l\right)}pr^{\left(l\right)}|l=1,2,\cdots ,\#F(pr)\}$, and *sub*^(*l*)^ represents the subscript of LT *F*^(*l*)^. The score function *E*(*F*(*pr*)) of *F*(*pr*) is given by

$$E(F(pr))=s_{\bar{\beta }}$$ (3)

where $\bar{\beta }=\sum_{l=1}^{\#F(pr)}\left(sub^{\left(l\right)}pr^{\left(l\right)}\right)/\sum_{l=1}^{\#F(pr)}pr^{\left(l\right)}$.

For any two PLTSs *F*_1_(*pr*_1_) and *F*_2_(*pr*_2_) :

1. If *E*(*F*_1_(*pr*_1_))>*E*(*F*_2_(*pr*_2_)), then *F*_1_(*pr*_1_) is superior to *F*_2_(*pr*_2_), denoted by *F*_1_(*pr*_1_)≻*F*_2_(*pr*_2_).
2. If *E*(*F*_1_(*pr*_1_))<*E*(*F*_2_(*pr*_2_)), then *F*_2_(*pr*_2_) is superior to *F*_1_(*pr*_1_), denoted by *F*_1_(*pr*_1_)≺*F*_2_(*pr*_2_).
3. If *E*(*F*_1_(*pr*_1_)) = *E*(*F*_2_(*pr*_2_)), then *F*_1_(*pr*_1_) is indifferent to *F*_2_(*pr*_2_), denoted by *F*_1_(*pr*_1_)~*F*_2_(*pr*_2_).

**Definition 9 [11].** Let a PLTS $F(pr)=\{F^{\left(l\right)}pr^{\left(l\right)}|l=1,2,\cdots ,\#F(pr)\}$. The score function *E*(*F*(*pr*)) of *F*(*pr*) is given by $E(F(pr))=s_{\bar{\beta }}$ with $\bar{\beta }=\sum_{l=1}^{\#F(pr)}\left(sub^{\left(l\right)}pr^{\left(l\right)}\right)/\sum_{l=1}^{\#F(pr)}pr^{\left(l\right)}$. The deviation degree of *F*(*pr*) is given by:

$$\bar{\rho }\left(F(pr)\right)={\left(\sum_{l=1}^{\#F(pr)}{\left(pr^{\left(l\right)}(sub^{\left(l\right)}-\bar{\beta })\right)}^{2}\right)}^{\frac{1}{2}}/\sum_{l=1}^{\#F(pr)}pr^{\left(l\right)}$$ (4)

For any two PLTSs *F*_1_(*pr*_1_) and *F*_2_(*pr*_2_) with *E*(*F*_1_(*pr*_1_)) = *E*(*F*_2_(*pr*_2_)):

- $\bar{\rho }(F_{1}(pr_{1}))>\bar{\rho }(F_{2}(pr_{2}))$, then *F*_2_(*pr*_2_) is superior to *F*_1_(*pr*_1_), expressed by *F*_1_(*pr*_1_)≺*F*_2_(*pr*_2_).
- $\bar{\rho }(F_{1}(pr_{1}))<\bar{\rho }(F_{2}(pr_{2}))$, then *F*_1_(*pr*_1_) is superior to *F*_2_(*pr*_2_), expressed by *F*_1_(*pr*_1_)≻*F*_2_(*pr*_2_).
- $\bar{\rho }(F_{1}(pr_{1}))=\bar{\rho }(F_{2}(pr_{2}))$, then *F*_1_(*pr*_1_) is equal to *F*_2_(*pr*_2_), expressed by *F*_1_(*pr*_1_)~*F*_2_(*pr*_2_).

**Definition 10 [61]:** Let $F_{1}(pr_{1})=\{F_{1}^{\left(l\right)}pr_{1}^{\left(l\right)}|l=1,2,\cdots ,\#F_{1}(pr_{1})\}$ and $F_{2}(pr_{2})=\{F_{2}^{\left(l\right)}pr_{2}^{\left(l\right)}|l=1,2,\cdots ,\#F_{2}(pr_{2})\}$ be any two normalized PLTSs. According to definitions 5, 6 and 7, the normalized PLTSs $F_{1}^{N}(pr_{1}^{N})$ and $F_{2}^{N}(pr_{2}^{N})$ are obtained. The distance between them is as follows:

$$d(F_{1}(pr_{1}),F_{2}(pr_{2}))=\sum_{l=1}^{\#F(pr)}pr(sub_{1}^{N(l)},sub_{2}^{N(l)})d(sub_{1}^{N(l)},sub_{2}^{N(l)})$$ (5)

In which $pr(sub_{1}^{N(l)},sub_{2}^{N(l)})=pr(sub_{1}^{N(l)})p(sub_{2}^{N(l)})=pr_{1}^{N(l)}pr_{2}^{N(l)}$, $d(sub_{1}^{N(l)},sub_{2}^{N(l)})=sub_{1}^{N(l)}-sub_{2}^{N(l)}/T$, and *T* represents the quantity of LTs in *S*.

### 3.2 Weighting criteria: PL-BWM-Entropy

**Fig 2:**
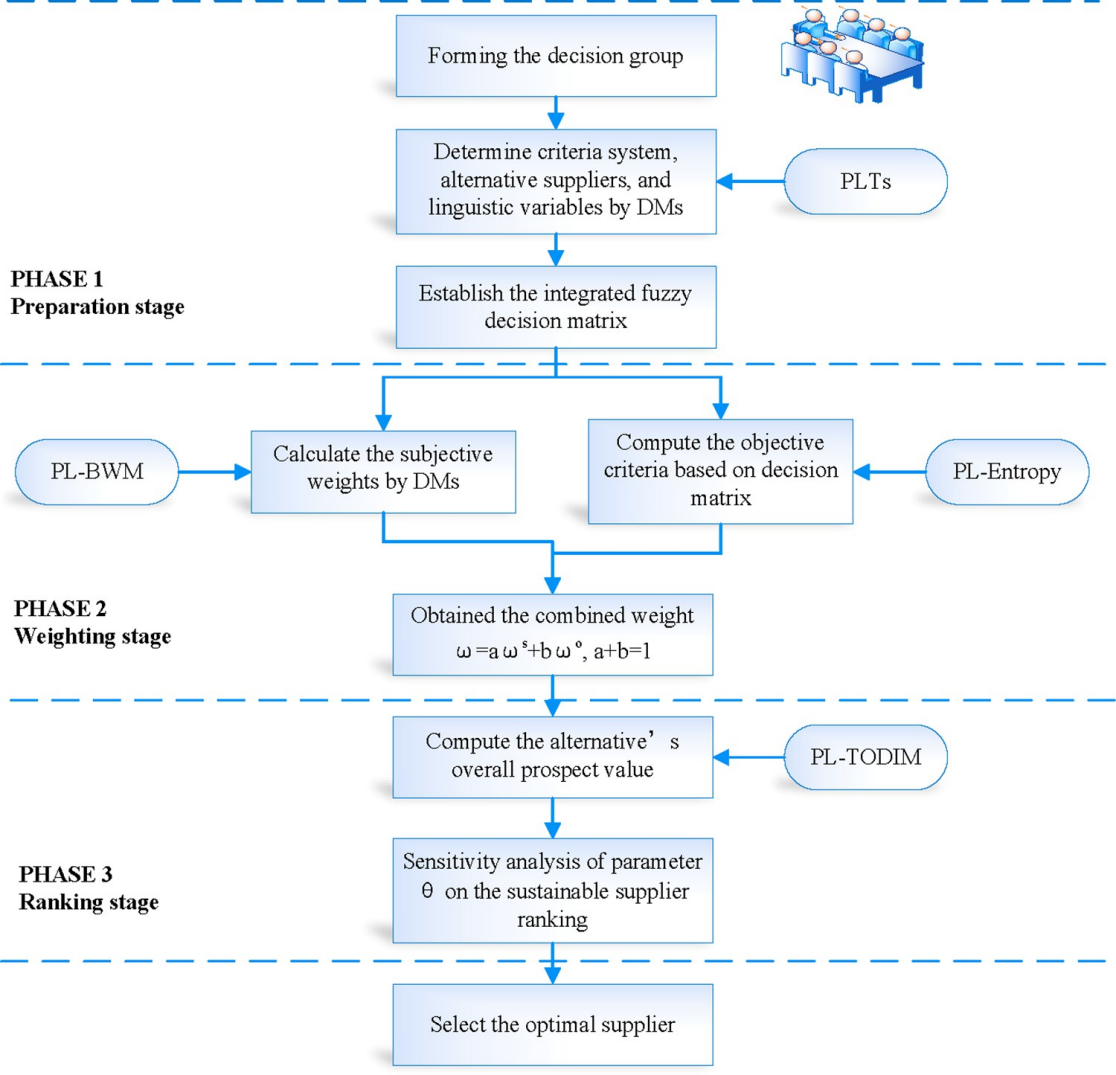
The conceptual framework of the MCGDM approach.

The MCGDM framework includes criteria weighting and suppliers ranking which is shown in Fig 2. In this framework, assigning the criteria weights is an important process, which is divided into subjective and objective weighting. The BWM, which simplifies the complex calculation process in terms of reference comparisons, is a subjective method. The entropy weight method is a typical objective approach in which the information entropy of each index is calculated according to a decision matrix.

The value of information entropy is inversely proportional to the final weight. The criteria weights related to comprehensive methods are more effective and stable.

### 3.2.1 The PL-BWM model

The BWM approach was presented by Rezaei, which includes selection, reference comparisons and computation steps. The calculation process of the PL-BWM approach are given by [62]:

**Step 1:** *C*_*j*_ = (*C*_1_,*C*_2_,⋯,*C*_*n*_) which denotes the set of criteria is selected by the DM, and the best criterion *C*_*B*_ and worst criterion *C*_*W*_ are also marked to represent the importance.

**Step 2:** The DM provides the preference of *C*_*B*_ over *C*_*j*_ and *C*_*j*_ over *C*_*W*_, which are expressed by PLTSs:

$$F^{Bj}(pr)=\{F^{Bj(l)}pr^{Bj(l)}|F^{Bj(l)}\in S,pr^{Bj(l)}\geq 0,l=1,2,\cdots ,\#F^{Bj}(pr),\sum_{i}^{\#F^{Bj}(pr)}pr^{Bj(l)}\leq 1\}$$ (6)

$$F^{jW}(pr)=\{F^{jW(l)}pr^{jW(l)}|F^{jW(l)}\in S,pr^{jW(l)}\geq 0,l=1,2,\cdots ,\#F^{jW}(pr),\sum_{i}^{\#F^{jW}(pr)}pr^{jW(l)}\leq 1\}$$ (7)

**Step 3:** The expected values of *F*^*Bj*^(*pr*) and *F*^*jW*^(*pr*) are calculated as follows:

$$\mu (F^{Bj}(pr))=\sum_{i=1}^{\#F^{Bj}(pr)}\left(f(F^{Bj(l)})\cdot pr^{Bj(l)}\right)$$ (8)

$$\mu (F^{jW}(pr))=\sum_{i=1}^{\#F^{jW}(pr)}\left(f(F^{jW(l)})\cdot pr^{jW(l)}\right)$$ (9)

where $\mu (F^{Bj}(pr^{Bj}))\in [0,1]$ and $\mu (F^{jW}(pr^{jW}))\in [0,1]$. In addition, *f* denotes a linguistic scale function, and the calculation results of *f* are between 0 and 1.


$$\left\{ \begin{array}{l} f(s_{l})=\left(l+\tau \right)/2\tau \\ f^{-1}(\theta_{l})=2\tau \cdot (\theta_{l}-\tau ) \end{array},if\;l\in \left[-\tau ,\tau \right]\right.$$ (10)

**Step 4:** Next, *A*_*B*_ shows the comparison between the *C*_*B*_ and other criteria, while *A*_*W*_ shows the comparison between other criteria and *C*_*W*_ using PLTSs:

$$A_{B}=(\mu (F^{B1}(pr)),\mu (F^{B2}(pr)),\cdots ,\mu (F^{Bn}(pr)))$$ (11)

$$A_{W}=(\mu (F^{1W}(pr)),\mu (F^{2W}(pr)),\cdots ,\mu (F^{nW}(pr)))$$ (12)

**Step 5:** Finally, the subjective criteria weight is calculated under the following linear programming model:

$$\begin{array}{l} \min\xi \\ s.t.\\ |w_{B}-\mu (F^{Bj}(pr))w_{j}^{s}|\leq \xi ,\;\mathrm{for}\;\mathrm{all}\;j\\ |w_{j}^{s}-\mu (F^{jW}(pr))w_{W}|\leq \xi ,\;\mathrm{for}\;\mathrm{all}\;j\\ \sum_{j}^{n}w_{j}^{s}=1\\ w_{j}\geq 0,\;\mathrm{for}\;\mathrm{all}\;j \end{array}$$ (13)

The final subjective criteria weight vector $w_{j}^{s}=(w_{1}^{s},w_{2}^{s},\cdots ,w_{n}^{s})$ is determined.

### 3.2.2 The PL-Entropy model

The entropy method is a dominant weight determination method based on the evaluation matrix given by the DMs [63]. We assume that all alternatives and criteria are *A*_*i*_ and *C*_*j*_. Under the environment of PLTSs, the specific steps of calculating the criteria weight by the entropy approach are given by [64]:

**Step 1:** After normalizing the decision matrix *S* = (*s*_*ij*_)_*m*×*n*_ by definitions 5, 6 and 7, the information entropy of the *j* th criterion is given by:

$$H_{j}=-\left[\sum_{i=1}^{m}\left(\frac{\sum_{l=1}^{\#F_{ij}(pr)}sub_{ij}{}^{\left(l\right)}pr_{ij}{}^{\left(l\right)}}{\#F_{ij}(pr)})\ln(\frac{\sum_{l=1}^{\#F_{ij}(pr)}sub_{ij}{}^{\left(l\right)}pr_{ij}{}^{\left(l\right)}}{\#F_{ij}(pr)}\right)\right]/\ln\;m$$ (14)

in which *m* represents the total number of alternatives.

**Step 2:** The criterion objective weight can be calculated by the obtained *H*_*j*_. The formula is as follows:

$$w_{j}^{o}=\frac{1-H_{j}}{n-\sum_{j=1}^{n}H_{j}}$$ (15)

**Step 3:** The criteria final in the basis of subjective and objective comprehensive weighting method are as below:

$$w_{j}=aw_{j}^{s}+bw_{j}^{o}\;a,b\in [0,1]$$ (16)

in which *a*+*b* =1.

### 3.3 Ranking suppliers: PL-TODIM model

The TODIM model proposed by Ma, Fan and Huang [63] on the basis of prospect theory. The alternatives assessment is established by capturing the bounded rationality of the human. This ranking result reflects the psychological characteristics of the DMs in avoiding risks. The calculation process is given by [64]:

**Step 1.** We need to convert all criteria (including benefit and cost types) into the same type by using Eq (17). Then, we normalize the decision matrix *S* = (*s*_*ij*_)_*m*×*n*_ into *R* = (*r*_*ij*_)_*m*×*n*_ by using definitions 5-7.


$$F_{ij}(pr_{ij})=\{\begin{array}{l} \{F_{ij}{}^{\left(d\right)}(pr_{ij}{}^{\left(d\right)})|d=1,2,\cdots ,\#F_{ij}(pr_{ij})\}\;\mathrm{for}\;\mathrm{benefit}\;\mathrm{criteria}\\ f^{-1}\left(\underset{\gamma_{ij}^{\left(d\right)}\in f(F_{ij})}{\cup }\left(1-\gamma_{ij}^{\left(d\right)}\right)\right)(pr_{ij}{}^{\left(d\right)})|d=1,2,\cdots ,\#F_{ij}(pr_{ij})\;\mathrm{for}\;\mathrm{cost}\;\mathrm{criteria} \end{array}$$ (17)

**Step 2.** The comprehensive weight of criteria *w*_*j*_ is obtained in Eq (16), and we can compute the relative weight *w*_*jp*_ according to:

$$w_{jp}^{}=\frac{w_{j}^{}}{w_{p}^{}}$$ (18)

in which *w*_*j*_ represents the weight of criterion *C*_*j*_, $w_{p}=\underset{j}{\max}\{w_{j}|j=1,2,\cdots ,n\}$.

**Step 3.** We compute the overall dominance degree *φ* of *A*_*i*_ over *A*_*o*_ corresponding to each criterion *C*_*j*_ as follows:

$$\varphi \left(A_{i},A_{o}\right)=\sum_{j=1}^{n}\phi_{j}\left(A_{i},A_{o}\right)$$ (19)

where

$$\phi_{j}(A_{i},A_{o})=\{\begin{array}{l} \sqrt{w_{jp}d(F_{ij}(pr_{ij})-F_{ij}(pr_{ej}))/\sum_{j=1}^{n}w_{jp}}\;\mathrm{if}\;F_{ij}(pr_{ij})≻F_{ij}(pr_{ej})\\ 0\;\mathrm{if}\;F_{ij}(pr_{ij})∼F_{ij}(pr_{ej})\\ -\frac{1}{\theta }\sqrt{\left(\sum_{j=1}^{n}w_{jp})d(F_{ij}(pr_{ij})-F_{ij}(pr_{ej})\right)/w_{jp}}\;\;\mathrm{if}F_{ij}(pr_{ij})≺F_{ij}(pr_{ej}) \end{array}$$ (20)

where *i*,*o* = 1,2,⋯,*m*. *θ* denotes the attenuation factor of the losses provided by DMs and *θ*>0. When $F_{ij}(pr_{ij})≻F_{ij}(pr_{ej})$, *ϕ*_*j*_(*A*_*i*_,*A*_*o*_) denotes a gain. If $F_{ij}(pr_{ij})≺F_{ij}(pr_{ej})$, *ϕ*_*j*_(*A*_*i*_,*A*_*o*_) represents a loss.

**Step 4.** According to the following form, we can calculate overall prospect value of each alternative *A*_*i*_.

$$\delta_{i}=\frac{\sum_{o=1}^{m}\varphi (A_{i},A_{o})-\underset{i}{\min}\{\sum_{o=1}^{m}\varphi (A_{i},A_{o})\}}{\underset{i}{\max}\{\sum_{o=1}^{m}\varphi (A_{i},A_{o})\}-\underset{i}{\min}\{\sum_{o=1}^{m}\varphi (A_{i},A_{o})\}},i=1,2,\cdots ,m$$ (21)

where higher values of *δ*_*i*_ indicate that alternative *A*_*i*_ is better.

### 3.4 Formulating the mathematical model

The proposed MOO model considers multiple suppliers and products comprehensively and aims to assign the final order quantity to different sustainable suppliers with minimize costs and CO*_2_* emissions, maximize the purchase value.

The relevant indices, parameters, variables and mathematical models are as follows.

**Table:** 

**Sets and indices**
M: Set of suppliers, M= {1, 2, …, m}
P: Set of products, P= {1, 2, …, p}
i: Supplier indices, where i∈M
t: Product indices, where t∈M
**Parameter**
$C_{it}^{p}$: Unit purchasing cost of product t ordered from suppler i
$C_{it}^{r}$: Unit transportation cost per mile of product t ordered from suppler i
$C_{it}^{a}$: Unit administration cost of product t ordered from suppler i
*W*_*i*_: weight of the supplier i
*d*_*i*_: transportation distance(mile) of product from supplier i
*P*_*it*_: maximum capacity (units)of product t from supplier i
*U*_*it*_: minimum quantity (units) of product t ordered from supplier i
CT: transportation capacity (units) per truck
*CO*_2*i*_: CO2 emissions (gram) per mile during truck driving from supplier i
**Decision Variables**
*q*_*it*_: quantity to be ordered from supplier i for product t
$y_{it}:\{\begin{array}{l} 1;\;if\;supplier\;iis\;selected\;for\;productt\\ 0;\;otherwise \end{array}$

model

$$\max\;Z_{1}=\sum_{i}\sum_{t}\left(W_{i}q_{it}\right)$$ (22)

$$\min\;Z_{2}=\sum_{i}\sum_{t}\left(C_{it}^{p}q_{it}+C_{it}^{a}y_{it}+C_{it}^{r}d_{i}q_{it}/CT\right)$$ (23)

$$\min\;Z_{3}=\sum_{i}\sum_{t}\left(CO_{2i}q_{it}d_{i}/CT\right)$$ (24)

subject to

$$\sum_{i}\sum_{t}q_{it}\leq P_{it}y_{it}$$ (25)

$$\sum_{i}\sum_{t}q_{it}\geq U_{it}$$ (26)

$$q_{it}\geq 0\;\forall i,t$$ (27)

$$y_{it}\in \{1,0\}\;\forall i,t$$ (28)

The objective functions in (22), (23), and (24) represent the maximization of the total purchase value, the minimization of the total cost, and the minimization of carbon dioxide emissions during transportation, respectively. (24) effectively reflects the sustainability of the supplier in the order allocation process. Constraint (25) ensures that the order quantity from supplier i should be within its supply capacity. In constraint (26), the order quantity is required to be greater than the minimum order quantity specified by supplier i. Constraint (27) limits nonnegative ordered quantity. Constraint (28) denotes sign constraint.

### 3.4.1 Constructing the FMOO model

Fluctuations in the supply chain will cause uncertainty in various costs. To deal with the dynamic nature of purchasing costs, transportation costs, management costs, and carbon dioxide emissions in the proposed model, we introduce fuzzy logic into the trapezoidal MOO model. The specific FMOO model is calculated as below [30, 31]:

$$\max Z_{1}=\sum_{i}\sum_{t}\left(W_{i}q_{it}\right)$$ (29)

$$\min Z_{2}=\sum_{i}\sum_{t}\left[(\frac{C_{it}^{p1}+2C_{it}^{p2}+2C_{it}^{p3}+C_{it}^{p4}}{6})q_{it}+(\frac{C_{it}^{a1}+2C_{it}^{a2}+2C_{it}^{a3}+C_{it}^{a4}}{6})y_{it}+(\frac{C_{it}^{r1}+2C_{it}^{r2}+2C_{it}^{r3}+C_{it}^{r4}}{6})d_{i}q_{it}/CT\right]$$ (30)

$$\min\;Z_{3}=\sum_{i}\sum_{t}(\frac{CO_{2i}^{1}+2CO_{2i}^{2}+2CO_{2i}^{3}+CO_{2i}^{4}}{6})q_{it}d_{i}/CT$$ (31)

subject to

$$\sum_{i}\sum_{t}q_{it}\leq [\frac{\alpha }{2}\cdot \frac{P_{it1}+P_{it2}}{2}+(1-\frac{\alpha }{2})\cdot \frac{P_{it3}+P_{it4}}{2}]y_{it}$$ (32)

$$\sum_{i}\sum_{t}q_{it}\geq [\frac{\alpha }{2}\cdot \frac{U_{it1}+U_{it2}}{2}+(1-\frac{\alpha }{2})\cdot \frac{U_{it3}+U_{it4}}{2}]$$ (33)

$$q_{it}\geq 0\;\forall i,t$$ (34)

$$y_{it}\in \{1,0\}\;\forall i,t$$ (35)

The confidence value *α*(0≤*α*≤1) provided by the DMs is represented in the above model. The superscripts 1-4 of the fuzzy parameters indicate the most pessimistic, the most likely and the most optimistic values [5]. For example, $C_{it}^{p1}$ and $C_{it}^{p4}$ represent the lowest and highest possible costs, respectively, for purchasing *t* products from supplier *i*. $C_{it}^{p2}$ and $C_{it}^{p3}$ together represent the two most likely purchasing costs for *t* from supplier *i*.

The linear membership function of (29) is given by:

$$\mu_{Z_{a}}=\{\begin{array}{l} 1;\;Z\leq Z_{\min}\\ \frac{Z_{\max}-Z}{Z_{\max}-Z_{\min}};\;Z_{\min}\leq Z\leq Z_{\max}\\ 0;\;Z\geq Z_{\max} \end{array}$$ (36)

The linear membership function of (30) and (31) are given by:

$$\mu_{Z_{b}}=\{\begin{array}{l} 1;\;Z\leq Z_{\max}\\ \frac{Z-Z_{\min}}{Z_{\max}-Z_{\min}};\;Z_{\min}\leq Z\leq Z_{\max}\\ 0;\;Z\geq Z_{\min} \end{array}$$ (37)

### 3.4.2 The augmented ε-constraint (AUGMECON) method

The traditional ε-constraint cannot guarantee the efficiency of the solution and requires a large computational load and long computation time when solving multiple objective functions. Mavrotas [65] proposed the augmented ε-constraint (AUGMECON) which produces a weak Pareto optimal solution and speeds up the calculation process, to overcome the disadvantages of the traditional ε-constraint.

When using AUGMECON, FMOO is converted into (38)–(41). Where (38) is the only objective function.

$$\max(Z_{1}+\eta \times S)$$ (38)

subject to

$$Z_{2}+S_{2}=\varepsilon_{2}$$ (39)

$$Z_{3}+S_{3}=\varepsilon_{3}$$ (40)

S≥0 (41)

subject to Eqs (32)–(35)

where S represents the surplus variable and *η* is within the range of [10^-3^, 10^-6^] commonly.

### 3.4.3 LP-metrics method

The LP-metrics method converts multiple objective functions in mathematical programming into a single objective function through the following formula [32]:

$$Min\;Z=[w_{1}^{Z}\frac{Z_{1}-Z_{1}^{*}}{Z_{1}^{*}}+w_{2}^{Z}\frac{Z_{2}-Z_{2}^{*}}{Z_{2}^{*}}+w_{3}^{Z}\frac{Z_{3}-Z_{3}^{*}}{Z_{3}^{*}}]$$ (42)

subject to Eqs (32)–(35) where $Z_{1}^{*},\;Z_{2}^{*}\;\mathrm{and}\;Z_{3}^{*}$ denote the ideal values of the objective functions and $w_{1}^{Z},\;w_{2}^{Z}\;\mathrm{and}\;w_{3}^{Z}$ are the weights assigned to the objective functions by the DMs to find more Pareto solution sets of FMOO through different weight combinations.

### 3.5 Selecting the final solution by TOPSIS

As a classic MCGDM method, TOPSIS selects the final solution by measuring the distance between the alternative and the optimal/worst alternatives. The detailed process is as below [66]:

Step 1. Normalize objective function value by following:

$${\bar{x}}_{ij}=\frac{x_{ij}}{\sqrt{\sum_{i=1}^{n}x_{ij}^{2}}}$$ (43)

Step 2: The objective function values ${\bar{x}}_{ij}$ with the integrated weights are given by:

$$v_{ij}=w_{j}^{Z}{\bar{x}}_{ij}$$ (44)

where $w_{j}^{Z}$ represents weight of the jth objective function.

Step 3: The separation measures for each alternative are obtained by:

$$S_{i}^{+}=\sqrt{\sum_{j=1}^{m}{\left(v_{j}^{+}-v_{ij}\right)}^{2}}$$ (45)

$$S_{i}^{-}=\sqrt{\sum_{j=1}^{m}{\left(v_{j}^{-}-v_{ij}\right)}^{2}}$$ (46)

in which $v_{j}^{+}$ and $v_{j}^{-}$ represent the PIS and NIS for each function, respectively.

Step 4: The closeness coefficient is given by:

$$CC_{i}=\frac{S_{i}^{-}}{S_{i}^{-}+S_{i}^{+}}$$ (47)

where a higher *CC*_*i*_ value corresponds to a better alternative *i*.

## 4 Illustrative example

We will give examples to prove the effectiveness of the proposed MCGDM-FMOO approach. To fulfill its social responsibility, company M will appoint three DMs (*d*_1_,*d*_2_,*d*_3_) to evaluate and select three sustainable suppliers (*A*_1_,*A*_2_,*A*_3_). Through reviewing the literature, we have constructed 4 economic criteria (*C*_1_), 4 environmental criteria (*C*_2_) and 3 social criteria (*C*_3_) [5, 30]. The details are represented in Table 3.

**Table 3:** Criteria system for SSS.

Criteria	Sub-criteria
Economic (*c*_1_)	Cost(*c*_11_)
	Product quality(*c*_12_)
	Technology capability(*c*_13_)
	Flexibility(*c*_14_)
Environmental(*c*_2_)	Environmental management systems(*c*_21_)
	Pollution control(*c*_22_)
	Energy consumption(*c*_23_)
	Recycling(*c*_24_)
Social(*c*_3_)	Labor health and rights(*c*_31_)
	Staff development(*c*_32_)
	Information disclosure(*c*_33_)

All suppliers can provide enterprises with two types of products, a and b. According to the results of the ranking, the DMs weight the suppliers as (0.5, 0.3, 0.2). The transportation distances between the three suppliers and the company are (305 miles, 300 miles, and 310 miles). The supplier’s maximum production capacity and the company’s minimum order quantity are shown in Table 4.

**Table 4:** Maximum production capacity and minimum ordered quantity.

Suppliers	*P*_*it*1_	*P*_*it*2_	*P*_*it*3_	*P*_*it*4_
1	20000	21000	22000	23000
2	256000	26000	27000	28000
3	23000	24000	25000	26000
	*U*_*i*11_	*U*_*i*21_	*U*_*i*12_	*U*_*i*22_
1	2000	2100	2200	2300
2	3000	3100	3200	3300
3	1500	1600	1700	1800

The unit purchasing cost, the transportation cost and the administration cost are shown in Table 5. The logistics activities of all suppliers are outsourced to third-party logistics, so we believe that the transportation capacity of each truck is *CT* = 5000, and the CO_2_ emissions per mile during transportation by truck reach 35 g/mile.

**Table 5:** Unit purchasing/transportation/administration cost.

Supplier	$C_{ia}^{p1}$	$C_{ib}^{p1}$	$C_{ia}^{p2}$	$C_{ib}^{p2}$	$C_{ia}^{p3}$	$C_{ib}^{p3}$	$C_{ia}^{p4}$	$C_{ib}^{p4}$
1	1	1.2	1.1	1.3	1.2	1.4	1.3	1.5
2	0.9	1.1	1	1.2	1.1	1.3	1.2	1.4
3	1.1	1.05	1.2	1.15	1.3	1.25	1.4	1.35
	$C_{ia}^{a1}$	$C_{ib}^{a1}$	$C_{ia}^{a2}$	$C_{ib}^{a2}$	$C_{ia}^{a3}$	$C_{ib}^{a3}$	$C_{ia}^{a4}$	$C_{ib}^{a4}$
1	0.1	0.08	0.105	0.085	0.11	0.09	0.115	0.095
2	0.07	0.13	0.075	0.135	0.08	0.14	0.085	0.145
3	0.11	0.14	0.115	0.145	0.12	0.15	0.125	0.155
	$C_{ia}^{r1}$	$C_{ib}^{r1}$	$C_{ia}^{r2}$	$C_{ib}^{r2}$	$C_{ia}^{r3}$	$C_{ib}^{r3}$	$C_{ia}^{r4}$	$C_{ib}^{r4}$
1	0.03	0.12	0.031	0.121	0.032	0.122	0.033	0.123
2	0.05	0.14	0.051	0.141	0.052	0.142	0.053	0.143
3	0.04	0.011	0.041	0.012	0.042	0.013	0.043	0.014

### 4.1 Stage 1: Calculation of the criteria weights and supplier ranks by MCGDM approach

#### 4.1.1 Weighting the criteria

After constructing the criteria system, DMs use Eqs (6)–(16) in Section 3.2 to comprehensively weight the criteria. In PL-BWM, DMs assigned to "pollution control" (*C*_22_) was the most critical criterion and "staff development" (*C*_32_) was the least critical criterion based on negotiation of decision group. The evaluation process is shown in the following two matrices. the three DMs jointly executed the reference comparisons with PLTS after consultation, Tables 6 and 7 show the fuzzy preferences of Best-to-Others and Others-to-Worst based on Table 8.

**Table 6:** The linguistic label for fuzzy preferences of the best criterion over all criteria.

Criteria		*c*_11_	*c*_12_	*c*_13_	*c*_14_	*c*_21_	*c*_22_	*c*_23_	*c*_24_	*c*_31_	*c*_32_	*c*_33_
Best Criterion	*l*_0_	0.6					1					
	*l*_1_	0.4	0.7			1						
	*l*_2_		0.3					0.2		0.55		
	*l*_3_							0.5		0.45		
	*l*_4_				0.1			0.3				
	*l*_5_			0.3	0.6				0.3			0.1
	*l*_6_			0.4	0.3				0.7			0.9
	*l*_7_			0.3							1	

**Table 7:** The linguistic label for fuzzy preferences of all criteria over the worst criterion.

Criteria	Worst Criterion							
	*l*_0_	*l*_1_	*l*_2_	*l*_3_	*l*_4_	*l*_5_	*l*_6_	*l*_7_
*c*_11_							0.4	0.6
*c*_12_						0.3	0.7	
*c*_13_	0.3	0.4	0.3					
*c*_14_		0.3	0.6	0.1				
*c*_21_							1	
*c*_22_								1
*c*_23_					0.3	0.5	0.2	
*c*_24_		0.7	0.3					
*c*_31_					0.45	0.55		
*c*_32_	1							
*c*_33_			0.1					

**Table 8:** Linguistic terms and corresponding degree in PLTS.

Linguistic terms	Corresponding degree
L_0_	None
L_1_	Worse
L_2_	Deficient
L_3_	Medium
L_4_	Above Average
L_5_	Adequate
L_6_	Impressive
L_7_	Outstanding

Then, we can transfer the fuzzy preferences showed in Tables 6 and 7 into matrices as follows:

$${\left\{F^{Bj}(p)\right\}}_{1\times 12}=[\begin{array}{l} \overset{C_{11}}{\left\{l_{0}(0.6),l_{1}(0.4)\right\}}\;\overset{C_{12}}{\left\{l_{1}(0.7),l_{2}(0.3)\right\}}\;\overset{C_{13}}{\left\{l_{5}(0.3),l_{6}(0.4),l_{7}(0.3\}\right\}}\;\overset{C_{14}}{\left\{l_{4}(0.1),l_{5}(0.6),l_{6}(0.3)\right\}}\;\overset{C_{21}}{\left\{l_{1}(1)\right\}}\\ \overset{C_{22}}{\left\{l_{0}(1)\right\}}\;\overset{C_{23}}{\left\{l_{2}(0.2),l_{3}(0.5),l_{4}(0.3)\right\}}\;\overset{C_{24}}{\left\{l_{5}(0.3),l_{6}(0.7)\right\}}\;\overset{C_{31}}{\left\{l_{2}(0.55),l_{3}(0.45)\right\}}\;\overset{C_{32}}{\left\{l_{7}(1)\right\}}\;\overset{C_{33}}{\left\{l_{5}(0.1),l_{6}(0.9)\right\}} \end{array}]$$

$${\left\{F^{jW}(p)\right\}}_{12\times 1}=[\begin{array}{l} \overset{C_{11}}{\left\{l_{6}(0.4),l_{7}(0.6)\right\}}\;\overset{C_{12}}{\left\{l_{5}(0.3),l_{6}(0.7)\right\}}\;\overset{C_{13}}{\left\{l_{0}(0.3),l_{1}(0.4),l_{2}(0.3)\right\}}\;\overset{C_{14}}{\left\{l_{1}(0.3),l_{2}(0.6),l_{3}(0.1)\right\}}\;\overset{C_{21}}{\left\{l_{6}(1)\right\}}\\ \overset{C_{22}}{\left\{l_{7}(1)\right\}}\;\overset{C_{23}}{\left\{l_{4}(0.3),l_{5}(0.5),l_{6}(0.2)\right\}}\;\overset{C_{24}}{\left\{l_{1}(0.7),l_{2}(0.3)\right\}}\;\overset{C_{31}}{\left\{l_{4}(0.45),l_{5}(0.55)\right\}}\;\overset{C_{32}}{\left\{l_{0}(1)\right\}}\;\overset{C_{33}}{\left\{l_{1}(0.9),l_{2}(0.1)\right\}} \end{array}]$$

Through Eqs (8)–(10), the following specific linear programming model are constructed based on Eq (13). The final subjective weight $w_{j}^{s}$ are obtained by solving the linear programming in Lingo 11.0. Finally, the $w_{j}^{s}$ are listed in Table 10.


$$\begin{array}{l} \min\xi \\ s.t.\\ |w_{B}-0.53w_{11}^{s}|\leq \xi ,|w_{B}-0.7w_{12}^{s}|\leq \xi ,|w_{B}-0.84w_{13}^{s}|\leq \xi ,|w_{B}-0.87w_{14}^{s}|\leq \xi ,\\ |w_{B}-0.57w_{21}^{s}|\leq \xi ,|w_{B}-0.5w_{22}^{s}|\leq \xi ,|w_{B}-0.58w_{23}^{s}|\leq \xi ,|w_{B}-0.67w_{24}^{s}|\leq \xi ,\\ |w_{B}-0.67w_{31}^{s}|\leq \xi ,|w_{B}-1w_{32}^{s}|\leq \xi ,|w_{B}-0.92w_{33}^{s}|\leq \xi ,\\ |w_{11}^{s}-0.97w_{W}|\leq \xi ,|w_{12}^{s}-0.91w_{W}|\leq \xi ,|w_{13}^{s}-0.57w_{W}|\leq \xi ,|w_{14}^{s}-0.63w_{W}|\leq \xi ,\\ |w_{21}^{s}-0.93w_{W}|\leq \xi ,|w_{22}^{s}-1w_{W}|\leq \xi ,|w_{23}^{s}-0.85w_{W}|\leq \xi ,|w_{24}^{s}-0.59w_{W}|\leq \xi ,\\ |w_{31}^{s}-0.83w_{W}|\leq \xi ,|w_{32}^{s}-0.5w_{W}|\leq \xi ,|w_{33}^{s}-0.58w_{W}|\leq \xi \\ w_{11}^{s}+w_{12}^{s}+w_{13}^{s}+w_{14}^{s}+w_{21}^{s}+w_{22}^{s}+w_{23}^{s}+w_{24}^{s}+w_{31}^{s}+w_{32}^{s}+w_{33}^{s}=1\\ w_{11}\geq 0,w_{12}\geq 0,w_{13}\geq 0,w_{14}\geq 0,w_{21}\geq 0,w_{22}\geq 0,w_{23}\geq 0,w_{24}\geq 0,w_{31}\geq 0,w_{32}\geq 0,w_{33}\geq 0 \end{array}$$

PL-Entropy is used for objective weighting. First, we integrate the evaluation matrix according to the DM’s opinions based on Table 8 and normalize the matrix as shown in Table 9. Then, the objective weights $w_{j}^{o}$ are obtained by Eqs (14)–(15). Integrating $w_{j}^{s}$ and $w_{j}^{s}$ into the comprehensive weight *w_j_* is the final step of weighting by Eq (16). Table 10 present subjective, objective and comprehensive criteria weights. In the weighting process, it is found that “technological capability” (0.05) and “energy consumption” (0.126) are regarded as the least important and most important practices in SSS.

**Table 9:** Normalized group decision matrix in PLTS.

Criteria	*A*_1_	*A*_2_	*A*_3_
Cos	$\left\{l_{3}(0.6),l_{4}(0.3),l_{2}(0.1)\right\}$	$\left\{l_{3}(0.4),l_{2}(0.4),l_{1}(0.2)\right\}$	$\left\{l_{5}(0.7),l_{7}(0.2),l_{6}(0.1)\right\}$
Pro	$\left\{l_{4}(0.7),l_{3}(0.2),l_{5}(0.1)\right\}$	$\left\{l_{3}(0.4),l_{4}(0.3),l_{2}(0.3)\right\}$	$\left\{l_{5}(0.4),l_{6}(0.3),l_{4}(0.3)\right\}$
Tec	$\left\{l_{3}(0.5),l_{4}(0.2),l_{2}(0.3)\right\}$	$\left\{l_{2}(0.4),l_{1}(0.5),l_{0}(0.1)\right\}$	$\left\{l_{4}(0.3),l_{2}(0.5),l_{3}(0.2)\right\}$
Fle	$\left\{l_{3}(0.5),l_{2}(0.3),l_{1}(0.2)\right\}$	$\left\{l_{3}(0.6),l_{5}(0.3),l_{4}(0.1)\right\}$	$\left\{l_{3}(0.6),l_{5}(0.2),l_{4}(0.2)\right\}$
Env	$\left\{l_{6}(0.5),l_{5}(0.4),l_{4}(0.1)\right\}$	$\left\{l_{3}(0.5),l_{4}(0.3),l_{5}(0.2)\right\}$	$\left\{l_{3}(0.6),l_{2}(0.3),l_{1}(0.1)\right\}$
Pol	$\left\{l_{5}(0.5),l_{3}(0.3),l_{4}(0.2)\right\}$	$\left\{l_{4}(0.7),l_{6}(0.2),l_{5}(0.1)\right\}$	$\left\{l_{4}(0.5),l_{5}(0.3),l_{3}(0.2)\right\}$
Ene	$\left\{l_{7}(0.4),l_{5}(0.4),l_{6}(0.2)\right\}$	$\left\{l_{6}(0.6),l_{5}(0.3),l_{7}(0.1)\right\}$	$\left\{l_{2}(0.3),l_{1}(0.1),l_{0}(0.6)\right\}$
Rec	$\left\{l_{6}(0.5),l_{4}(0.3),l_{5}(0.2)\right\}$	$\left\{l_{6}(0.7),l_{5}(0.2),l_{7}(0.1)\right\}$	$\left\{l_{3}(0.7),l_{1}(0.2),l_{2}(0.1)\right\}$
Lab	$\left\{l_{2}(0.3),l_{3}(0.2),l_{1}(0.5)\right\}$	$\left\{l_{3}(0.6),l_{4}(0.3),l_{2}(0.1)\right\}$	$\left\{l_{5}(0.4),l_{3}(0.4),l_{4}(0.2)\right\}$
Sta	$\left\{l_{3}(0.4),l_{4}(0.2),l_{2}(0.4)\right\}$	$\left\{l_{4}(0.3),l_{3}(0.3),l_{2}(0.4)\right\}$	$\left\{l_{3}(0.4),l_{4}(0.3),l_{2}(0.3)\right\}$
Inf	$\left\{l_{4}(0.8),l_{5}(0.1),l_{3}(0.1)\right\}$	$\left\{l_{4}(0.7),l_{3}(0.2),l_{5}(0.1)\right\}$	$\left\{l_{3}(0.3),l_{1}(0.5),l_{2}(0.2)\right\}$

**Table 10:** Subjective weight, objective weight, comprehensive weight and relative weight of sub-criteria *c*_*j*_.

*c*_*j*_	$w_{j}^{s}$	$w_{j}^{o}$	*w*_*j*_	*w*_23*p*_
*c*_11_	0.116	0.094	0.105	0.83
*c*_12_	0.109	0.105	0.107	0.85
*c*_13_	0.07	0.029	**0.05**	0.396
*c*_14_	0.077	0.063	0.07	0.56
*c*_21_	0.111	0.104	0.107	0.85
*c*_22_	0.119	0.12	0.119	0.94
*c*_23_	0.094	0.157	**0.126**	1
*c*_24_	0.072	0.148	0.11	0.87
*c*_31_	0.099	0.06	0.079	0.63
*c*_32_	0.062	0.052	0.057	0.45
*c*_33_	0.071	0.068	0.069	0.55

### 4.1.2 Ranking the suppliers

After getting the criteria weights, DMs rank the suppliers by Eqs (17)–(21). *C*_23_ is the most critical criterion (*w*_23_ = 0.126). Hence, the weights *w*_23*p*_ of all criteria relative to *C*_23_ are presented below.


$$\begin{array}{l} w_{11P}=0.83,w_{12P}=0.85,w_{13P}=0.396,w_{14P}=0.56,w_{21P}=0.85,\\ w_{22P}=0.94,w_{23P}=1,w_{24P}=0.87,w_{31P}=0.63,w_{32P}=0.45,w_{33P}=0.55, \end{array}$$

Then, calculate the dominance of each supplier *A*_*i*_ over each supplier *A*_*o*_ with respect to criteria *C*_*j*_. For example, in order to calculate *ϕ*_11_(*A*_1_,*A*_2_), *j* = 11, *i* = 1, *o* = 2. Firstly, we can calculate the score function *E*(*F*(*pr*)) according to Eq (3) to get the comparison results *F*_111_(*pr*_111_)≻*F*_111_(*pr*_211_). Then, the distance *d*(*F*_11_(*pr*_11_)−*F*_11_(*pr*_21_)) = 0.913 between *F*_11_(*pr*_11_) and *F*_11_(*pr*_21_) are compute by Eq (5). *w*_11*p*_ = 0.83, $\sum_{j=1}^{n}w_{jp}=7.926$. Finally, *ϕ*_11_(*A*_1_,*A*_2_) = 0.31 are obtained by Eq (20).

Thirdly, the overall dominance degrees *φ* are obtained and shown in the following matrix by Eq (21). Then, calculate the overall prospect value of each alternatives *A*_*i*_(*i* = 1,2,3) (suppose *θ* = 1), *δ*(*A*_1_) = 1, *δ*(*A*_2_) = 0.46, *δ*(*A*_3_) = 0. For example, $\delta (A_{2})=\frac{-26.3-(-34.1)}{-17.17-(-34.1)}=0.46$, where $\sum_{o=1}^{m}\varphi (A_{2},A_{o})=(-14.7)+0+(-11.6)=26.3$.


$$\varphi =[\begin{array}{cccc} & x_{1} & x_{2} & x_{3}\\ x_{1} & 0 & -9.7 & -8.01\\ x_{2} & -14.7 & 0 & -11.6\\ x_{3} & -17 & -17.1 & 0 \end{array}]$$

Finally, rank the three suppliers in according with the value of *δ*(*A*_*i*_)(*i* = 1,2,3). The larger the value of *δ*(*A*_*i*_), the better the scheme *A*_*i*_, and get *A*_1_≻*A*_2_≻*A*_3_.

### 4.1.3 Analyzing the effect of the parameter *θ*

In the PL-TODIM method, the influence of loss will increase when 0<*θ*<1. While *θ*>1 indicates that the influence of loss will decrease. Therefore, we believe that *θ* will affect the overall dominance degree [35]. We observe its influence on the ranking by changing the value of *θ* (0.25-100). Table 11 shows the results.

**Table 11:** Ranking results for different *θ*.

Suppliers	*θ* = 0.25		*θ* = 0.5		*θ* = 0.75		*θ* = 1		*θ* = 1.5	
	*δ*	order	*δ*	order	*δ*	order	*δ*	order	*δ*	order
*A*_1_	1	1	1	1	1	1	1	1	1	1
*A*_2_	0.29	2	0.39	2	0.44	2	0.48	2	0.52	2
*A*_3_	0	3	0	3	0	3	0	3	0	3
	*θ* = 2		*θ* = 3		*θ* = 10		*θ* = 50		*θ* = 100	
	*δ*	order	*δ*	order	*δ*	order	*δ*	order	*δ*	order
*A*_1_	1	1	1	1	1	1	1	1	1	1
*A*_2_	0.55	2	0.58	2	0.39	2	0.396	2	0.397	2
*A*_3_	0	3	0	3	0	3	0	3	0	3

**Fig 3:**
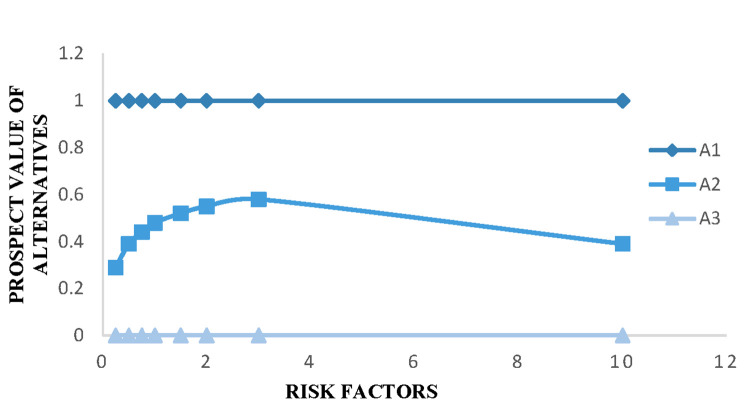
Alternatives’ prospect value of different θ-value for illustrative example.

As can been seen in Table 11, different values of *θ* between 0.25 and 100 will result in a different overall dominance degree. However, the supplier ranking always remains *A*_1_≻*A*_2_≻*A*_3_. Consequently, the results are consistent with the changed *θ*. Since the result caused by the change of *θ* is more sensitive in the range of 1-10, Fig 3 shows that the different risk factors lead to the change of three alternatives.

### 4.2 Stage 2: FMOO model

The FMOO is solved via Matlab2020a with the optimization toolbox running on a personal laptop Intel(R) Core (TM)i5-8250U CPU at 2.5 GHz with 8 GB of RAM and the Windows 10 operating system.

### 4.3 Stage 3: Using AUGMECON and LP-Metrics to solve the FMOO model

AUGMECON was originally used to solve the FMOO model. We convert Z1 to the objective function through Eq (38), and Z2 and Z3 are converted into constraints by Eqs (39)–(41). Here, *η* = 0.0001, and *ε* is assigned by DMs. LP-Metrics requires that the three objective functions be converted into a single objective function by Eq (42). The weights of different functions are considered in the mono-objective model, so the DMs assign the 20 weights of the original objective functions as shown in Table 12. This will allow us to obtain more Pareto solutions.

**Table 12:** Assigned weights of objective function in LP-metrics method.

#	Assigned weights	#	Assigned weights
	$w_{1}^{Z}$ $w_{2}^{Z}$ $w_{3}^{Z}$		$w_{1}^{Z}$ $w_{2}^{Z}$ $w_{3}^{Z}$
1	0.04,0.9,0.05	11	0.44,0.26,0.3
2	0.12,0.8,0.08	12	0.48,0.2,0.32
3	0.2,0.7,0.1	13	0.5,0.15,0.35
4	0.28,0.6,0.12	14	0.52,0.17,0.31
5	0.28,0.5,0.22	15	0.56,0.17,0.27
6	0.32,0.4,0.28	16	0.6,0.1,0.3
7	0.34,0.33,0.33	17	0.65,0.1,0.35
8	0.38,0.3,0.32	18	0.7,0.08,0.22
9	0.39,0.29,0.32	19	0.8,0.08,0.12
10	0.4,0.28,0.32	20	0.9,0.05,0.05

**Fig 4:**
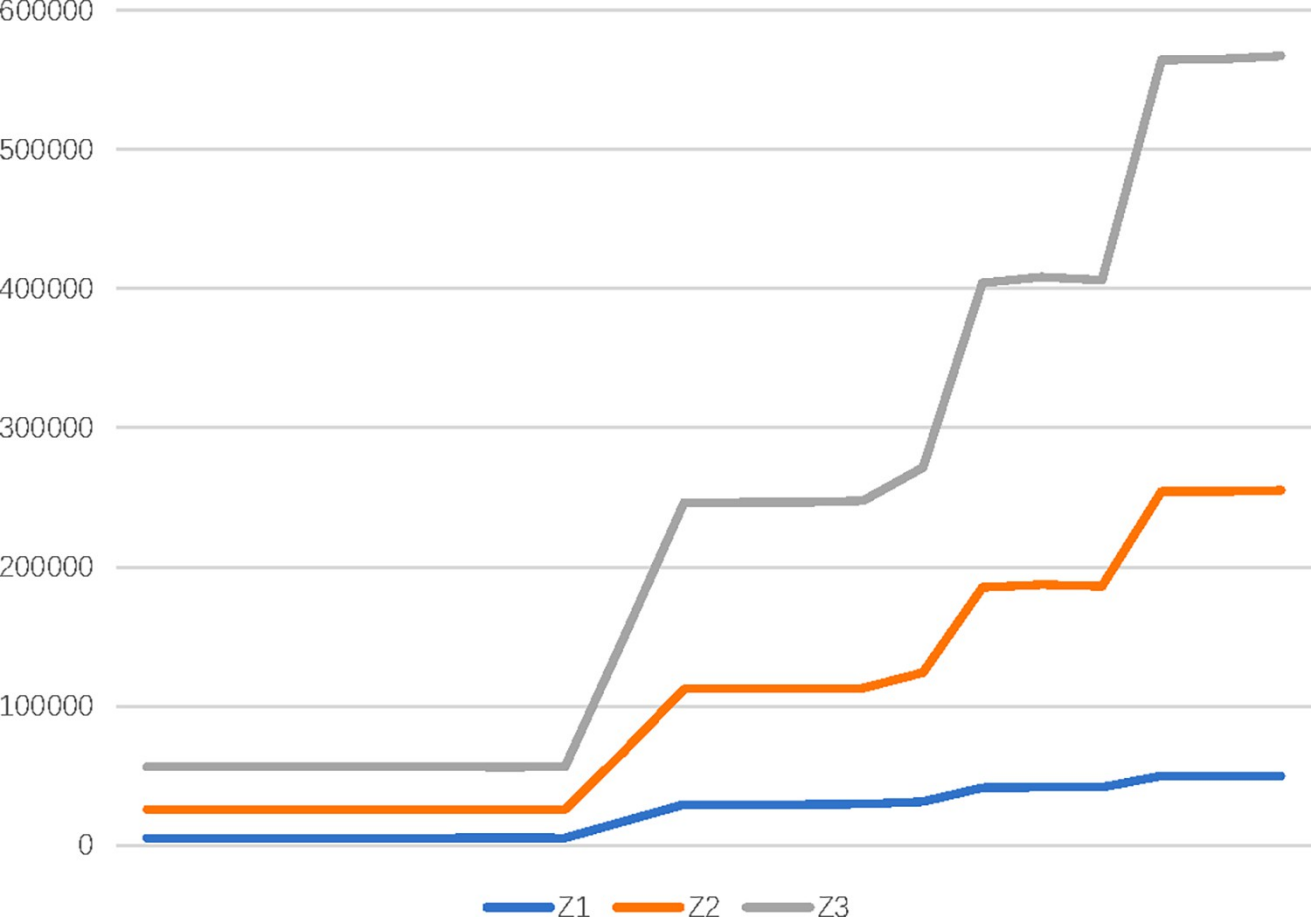
reto front with twenty weights for *α* = 0.1 of LP-Metrics method.

**Fig 5:**
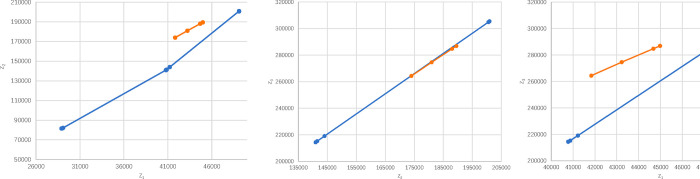
Pareto front for *α* = 0.5 of AUGMECON method.

Table 13 presents the objective values and order quantity by AUGMECON after ten α iterations, where *ε*_2_ = 184710 and *ε*_3_ = 299020. Similarly, Table 14 includes objective values and order quantities determined by LP-Metrics with ten α levels for $w_{1}^{Z}=0.9$, $w_{2}^{Z}=0.05$, and $w_{3}^{Z}=0.05$. For example, the quantity of *b* products that should be purchased from the first supplier was 27,165 when *α* = 0.1. In addition, Fig 4 presents the values of the objective functions after LP-Metrics runs 20 weight combinations. Therefore, we believe that as the weight of Z1 gradually increases, the company’s purchase value will not increase significantly, but the value of the total cost function and CO^2^ emission function will increase significantly. Fig 5 shows that when *α* = 0.5, the objective function values with 5 α levels are calculated by the above two approaches. Since the solutions obtained by the above two approaches are different, we take the results of both methods into account and provide more solutions for DMs.

**Table 13:** Results for AUGMECON approach.

#	*α*-level	maxZ_1_	minZ_2_	minZ_3_	*q*_1*a*_	*q*_1*b*_	*q*_2*a*_	*q*_2*b*_	*q*_3*a*_	*q*_3*b*_
1	0.1	45706	193550	291500	22400	25400	13420	27400	22400	25400
2	0.2	45523	191790	290340	22300	25300	13378	27300	22300	25300
3	0.3	45341	191020	289180	22200	25200	13336	27200	22200	25200
4	0.4	45158	190260	288020	22100	25100	13296	27100	22098	25100
5	0.5	44976	189500	286870	22000	25000	13253	27000	22000	25000
6	0.6	44793	188730	285710	21900	24900	13211	26900	21900	24900
7	0.7	44611	187970	284550	21800	24800	13169	26800	21800	24800
8	0.8	44428	187210	283390	21700	24700	13127	16700	21700	24700
9	0.9	44246	186440	282230	21600	24600	13085	26600	21600	24600
10	1	44063	185680	281070	21500	24500	13043	26500	21500	24500

**Table 14:** Results for LP-Metrics approach.

#	*α*-level	maxZ_1_	minZ_2_	minZ_3_	*q*_1*a*_	*q*_1*b*_	*q*_2*a*_	*q*_2*b*_	*q*_3*a*_	*q*_3*b*_
1	0.1	49900	205210	312050	27400	27165	21704	21450	24962	23392
2	0.2	49700	204240	310630	26976	27270	22299	21115	24203	23561
3	0.3	49499	202940	308300	27132	27001	21700	22168	24599	21761
4	0.4	49300	201570	306460	27044	27016	21715	22081	21590	24066
5	0.5	49100	200940	305480	26970	26947	21535	21641	23377	22565
6	0.6	48900	199930	303940	26878	26831	21502	21719	22632	22763
7	0.7	48699	198890	302510	26790	26790	21498	21439	21408	23732
8	0.8	48500	198060	301050	26698	26611	21512	21597	22363	22201
9	0.9	48300	197290	299950	26573	26545	21549	21182	22539	22071
10	1	48100	196330	298590	26474	26492	21420	21031	21720	22687

### 4.4 Stage 4: Selecting the solution by TOPSIS

Twenty-four effective Pareto solutions can be computed through AUGMECON and LP-Metrics to solve the FMOO model when *α* = 0.5 (Table 15). The DMs select the optimal solution by Eqs (43)–(47). Hence, we believe that the 21st solution is the optimal solution, and the corresponding order allocation is shown as Table 16:

**Table 15:** TOPSIS calculation result for *α* = 0.5.

#	Z_1_	Z_2_	Z_3_	Weighted normalized value			$S_{i}^{+}$	$S_{i}^{-}$	*CC_i_*
1	5221	19886	30386	0.0110	0.0113	0.0110	0.1435	0.0085	0.391
2	5206	19849	30325	0.0109	0.0113	0.0110	0.1436	0.0085	0.391
3	5202	19828	30293	0.0109	0.0113	0.0110	0.1436	0.0085	0.391
4	5204	19832	30299	0.0109	0.0113	0.0110	0.1436	0.0085	0.391
5	5205	19834	30303	0.0109	0.0113	0.0110	0.1436	0.0085	0.391
6	5202	19828	30293	0.0109	0.0113	0.0110	0.1436	0.0085	0.391
7	5205	19834	30303	0.0109	0.0113	0.0110	0.1436	0.0085	0.391
8	5617	20917	32089	0.0118	0.0119	0.0116	0.1427	0.0084	0.391
9	17116	49697	81168	0.0360	0.0284	0.0294	0.1212	0.0052	0.372
10	28890	81546	131440	0.0608	0.0465	0.0476	0.1053	0.0044	0.386
11	29004	81883	131980	0.0610	0.0467	0.0478	0.1052	0.0044	0.387
12	28997	81907	132010	0.0610	0.0467	0.0478	0.1052	0.0044	0.387
13	29062	82173	132390	0.0611	0.0469	0.0480	0.1051	0.0044	0.387
14	29078	82215	132460	0.0612	0.0469	0.0480	0.1051	0.0044	0.387
15	40774	140930	214370	0.0858	0.0804	0.0777	0.0886	0.0095	0.524
16	41219	144030	219060	0.0867	0.0822	0.0794	0.0882	0.0100	0.531
17	40866	141500	215210	0.0859	0.0807	0.0780	0.0886	0.0096	0.525
18	49095	200860	305340	0.1033	0.1146	0.1106	0.0923	0.0206	0.609
19	49100	200570	304860	0.1033	0.1144	0.1104	0.0923	0.0205	0.608
20	49100	200940	305480	0.1033	0.1146	0.1107	0.0923	0.0206	0.609
21	**44976**	**189500**	**286870**	**0.0946**	**0.1081**	**0.1039**	**0.0842**	**0.0181**	**0.615**
22	44677	188050	284770	0.0940	0.1073	0.1032	0.0837	0.0178	0.615
23	43222	181000	274590	0.0909	0.1033	0.0995	0.0815	0.0164	0.611
24	41831	173990	264360	0.0880	0.0993	0.0958	0.0800	0.0152	0.606

**Table 16:** The optimal order allocation from each supplier.

supplier	product *a* (unit)	product *b* (unit)
1	22,000	25,000
2	13,253	27,000
3	22,000	25,000

## 5 Result discussion and comparative analysis

In order to verify the effectiveness and novelty of the proposed model, this section mainly further discusses the results of Section 4, and conducts qualitative and quantitative comparative analysis.

### 5.1 Discussion

SSS/OA is a critical decision-making problem for contemporary enterprises. The proposed MCGDM-FMOO model takes into account comprehensive weights, criteria construction, ranking process, and multi-objective optimization. In the illustrated example, the PL-BWM-Entropy technique found "energy consumption" (0.126), "pollution control" (0.119), "recycling" (0.11), "product quality" (0.107), and "environmental management systems" (0.107) to be important practices in SSS, while "technological capability" (0.05) and "staff development" (0.057) are considered the least important practices. PL-TODIM considers psychological factors of decision process by parameter *θ*. However, we found that changes in the range of 0.25-100 did not affect the supplier ranking (*A*_1_≻*A*_2_≻*A*_3_), so we believe that PL-TODIM has strong robustness and consistency. Finally, we discussed the results of OA in three scenarios: (1) AUGMECON operation results with ten α levels (0.1-1) are shown in Table 13, (2) LP-Metrics operation results with ten α levels (0.1-1) for $w_{1}^{Z}=0.9$, $w_{2}^{Z}=0.05$, and $w_{3}^{Z}=0.05$ are shown in Table 14, and (3) LP-Metrics operation results with twenty objective weight combinations for *α* = 0.1 are shown in Fig 4. The results of (1) and (2) show that the Pareto solutions provided by the AUGMECON and LP-Metrics methods are different, but not significantly so. Therefore, it is believed that both results are reasonable, and they enrich the Pareto solution of the FMOO model. The results of (3) denote that the attitude of the DMs regarding Z1 will not significantly affect the purchasing value, and the trends of the three objective function values are not greatly affected by the weight change. In general, considering sustainability in supplier selection will lead to increased costs.

The proposed approach contributes the following advantages: (1) Within the MCGDM model, PL-BWM-Entropy combines linguistic operators and two weighting methods to minimize the loss of linguistic information. Moreover, the comprehensive weighting model can better integrate the evaluation opinions of DMs and objective evaluation information. So, it is more likely to provide accurate and comprehensive information than single method. (2) Under various situations of MCGDM problems, such as engineering, economy, management and military, it is necessary to rely on industry experts to make decisions. The PL-TODIM considers the loss aversion behavior of DMs, hence retains decision-making information of all DMs as much as possible under the premise of bounded rationality. (3) The proposed FMOO model can effectively depict the uncertainty of parameters by trapezoidal fuzzy number in four dimensions (pessimistic, optimistic and most likely values). As a popular method, AUGMECON denotes the augmented ε-constraint method, which reduces the computation load and computing time by improving the algorithm when solving FMOO. Then using AUGMECON and LP metrics provides a richer set of Pareto solutions of FMOO.

### 5.2 Qualitative comparison and analysis

The existing MCGDM methods can be roughly divided into three categories on the basis of their functions: 1. Weighting methods, such as AHP, ANP, BWM and Entropy method which are mainly used to weight criteria or DMs. 2. Ranking methods are utilized to sort the advantages and disadvantages of alternatives, including TOPSIS, VIKOR, TODIM, ELECTRE and PROMETHEE. Some weighting methods are also applicable to ranking, such as AHP. 3. Hybrid methods. Due to the complexity, a single method can’t solve the research problem. Therefore, the combined methods are proposed to deal with the defects of the single methods.

Many hybrid MCGDM methods for supplier selection have been developed. We selected some representative studies, from whether the OA problem was solved, whether the weight was calculated from the subjective and objective dimensions, whether the uncertainty in the decision-making process was handled, whether the psychological factors of DMs to avoid loss were considered, and the aggregation methods used in the literature were qualitatively compared with the proposed methods.

By qualitatively comparing the research scope of the literature in Table 17, the biggest highlight of this paper is that we consider the psychological factors of DMs to avoid losses, not just making decisions based on expected utility, and measure the criteria weight from both subjective and objective aspects. In addition, our model can express the degree of hesitation to avoid information loss as much as possible, which is not available in most other methods. More reasonable order allocation quantity can be obtained based on the results of SSS.

**Table 17:** Qualitative comparison of MCGDM techniques.

Literature	Optimal order quantity	Subjective &Objective	Handling uncertainty	Loss aversion	Aggregation method
Vahidi et al. [23]	Yes	No	No	No	SWOT-QFD
Divsalar et al. [67]	No	No	Yes	Yes	PHF-TODIM
Banaeian et al. [58]	No	No	Yes	No	TOPSIS-VIKOR-GRA
Gao et al. [68]	No	Yes	Yes	Yes	Cloud-TODIM
Song et al. [59]	No	No	Yes	No	DEMATEL
Jauhar and Pant [56]	No	No	Yes	No	DEA-DE-MODE
Our model	Yes	Yes	Yes	Yes	BWM-Entropy-TODIM

We selected some studies related to supplier selection and order allocation for comparison, including whether economic, environmental and social sustainability is involved in OA process, whether cost minimization, procurement value maximization and carbon dioxide emission minimization are included in the objective function, and whether the model can deal with multi product order allocation. Through the summary of order allocation in Table 18, compared with other literature, this paper fully considers the application of sustainability in the OA process, and effectively reflects environmental sustainability into the carbon dioxide emission function in the FMOO.

**Table 18:** Qualitative comparison of MCGDM techniques.

Literature	Order allocation							
	Sustainability			Objective function			Multi products	Approach
	Eco	Env	Soc	Total cost	Purchase value	Carbon emission		
Bektur. [30, 69]	√	√	√	√	√			FMOO
Moheb et al. [69]	√	√	√	√	√		√	MODM
Lo et al. [70]	√	√		√		√	√	FMOLP
Ghadimi. [71]	√	√	√	√			√	MODM
Mirzaee et al. [29]	√	√	√	√		√	√	MILP
Çebi and Otay [72]	√	√		√		√	√	FMOO
Our study	√	√	√	√	√	√	√	FMOO

### 5.3 Quantitative comparison and analysis

To demonstrate the effectiveness and novelty of the model more clearly, the proposed ranking model is quantitatively compared with other methods, and prove the effectiveness of this method by analyzing the similarities and differences of supplier ranking.

In order to control variables and keep consistent with the situation of MCDM problem, we firstly deal with the language rating in the two articles. The language level of PLTS is divided into seven levels in Tong et al [42]. In this paper, we divide linguistic variables into eight levels. By comparing the corresponding degree of the language sets, we believe that there is little difference in accuracy between the two articles. Therefore, when the PL-TODIM method in this paper is applied to the cases in [42], we believe that ignoring the ***l*_0_** (none) language set will not affect the final ranking. Finally, the corresponding relationship between the linguistic terms and corresponding degree in PLTS of the two articles is shown in the Table 19.

**Table 19:** Linguistic terms and corresponding degree in PLTS in two papers.

Linguistic variable	Worse	Deficient	Medium	Above Average	Adequate	Impressive	Outstanding
Tong et al. [42]	S_−3_	S_−2_	S_−1_	S_0_	S_1_	S_2_	S_3_
This paper	*l*_1_	*l*_2_	*l*_3_	*l*_4_	*l*_5_	*l*_6_	*l*_7_

Table C. 3 in reference [42] provides the comprehensive evaluation matrix expressed in PLTS. The evaluation of supplier A1 by the decision-making group under criterion C1 is $\left\{s_{1}(0.4),s_{2}(0.4),s_{3}(0.2)\right\}$. We convert this evaluation into $\left\{l_{5}(0.4),l_{6}(0.4),l_{7}(0.2)\right\}$ for convenience of calculation based on the Table 19.

Comparing different methods in the PLTS environment eliminates the error caused by language set. We compare PL-TODIM with PL-TOPSIS and PL-PROMETHEE II methods respectively, and the final ranking of five alternatives are presented in Table 20. In the final results, great changes have taken place in the ranking between PROMETHEE II method and the proposed method. We think the main reason for the large difference in ranking is that PROMETHEE method does not give expression to the psychological change process of DMs in the decision-making process although it fully considers the deviation of each supplier in the evaluation criteria. The ranking of PL-TODIM and PL-TOPSIS are similar. The best and worst alternatives are *A_3_* and *A_2_*. The opposite ranking of *A_1_* and *A_4_* may be due to the fact that TOPSIS is ranked according to the distance between best/worst and each alternative. The bounded rational behavior related to the individuals are also not considered. Instead, TODIM method reflects the psychological behavior of DMs, especially the degree to which different DMs avoid losses, which makes the decision-making better reflect the way of human thinking in reality. We believe that difference of ranking results caused by different principles of methods and the degree of considering the psychological factors of DMs. Therefore, we still believe that the proposed method in this paper is feasible and innovative.

**Table 20:** The results based on the comparing methods.

Ranking method	A1	A2	A3	A4	A5	Ranking
PL-TOPSIS	0.27	0.26	0.33	0.28	0.29	$A_{3}≻A_{5}≻A_{1}≻A_{4}≻A_{2}$
Classical PROMETHEE II	0.05	0.27	-0.34	0.16	-0.13	$A_{2}≻A_{4}≻A_{1}≻A_{5}≻A_{3}$
PL-PROMETHEE II	0.01	0.14	-0.18	0.07	-0.04	$A_{2}≻A_{4}≻A_{1}≻A_{5}≻A_{3}$
PL-TODIM	0.53	0	1	0.77	0.87	$A_{3}≻A_{5}≻A_{4}≻A_{1}≻A_{2}$

## 6 Conclusions and managerial implications

### 6.1 Research summary

Reasonable SSS/OA processes are an effective means to improve supply chain performance and are gradually becoming increasingly valued. In this study, we construct a TBL-based SSS criteria system and propose the hybrid MCGDM-FMOO approach to deal with the SSS/OA problem. Criteria are weighted by PL-BWM-Entropy from both a subjective and an objective perspective, and then suppliers are ranked considering the psychology of the DMs using the PL-TODIM method in the developed MCGDM framework. The goal of the OA problem is to reasonably allocate multiple products to multiple suppliers under the premise of maximizing purchase value, minimizing costs and minimizing CO_2_ emissions, as shown in the FMOO model. Here, AUGMECON and LP-Metrics provide more Pareto solutions as tools to solve FMOO. Then, TOPSIS can choose the solution that are closest to PIS and farthest from NIS, which we call the ideal solution. Finally, validity and practicability of solving the SSS/OA problems is proved by applying the MCGDM-FMOO model to an illustrative example. The method proposed can also be used to solve SSS/OA problems in other industries.

### 6.2 Managerial implications

MCGDM method has been widely used as the decision-making framework of intelligent system and industry decision-making [73]. This study has the following Managerial implications for decision-makers in practice: (1) Make full use of the experience and knowledge of DM group. The proposed method can better translate the uncertainty and hesitation of DMs into visual data in the PLTS environment. In addition, the evaluation information of all DMs can be retained without considering the group size. (2) Make the decision result more reasonable. In the decision-making system, the advantages and disadvantages of alternatives are obtained by evaluate multiple criteria. So, weighting criteria and alternatives ranking are two critical processes. The combined method of subjective and objective can better eliminate the impact of unreasonable criteria weight on the final decision. In the process of ranking, the bounded rationality of DMs is taken as the premise. As a result, these two aspects make the decision-making process more reasonable and credible. (3) Provide support for enterprises to improve and reconfigure the SCM process. The FMOO model established in this paper considers the total cost and environmental factors, it makes the order distribution in line with the current concept of sustainability on the premise of maximizing the profits. It is worth noting that multiple conflicting objectives cannot be achieved at the same time in the order allocation model, but the computing methods proposed in this paper can calculate the results of different weight allocation among multiple objectives, so as to provide more feasible solutions for the actual situation. In addition, the objective function of maximum purchasing value is established based on the SSS results, which effectively connects the SSS and OA problems, and makes the two independent functions in supply chain management become a coherent series of decision-making activities. The coherent activities reduce the probability that the organization will lose profits due to lack of coordination. (4) Provides universality and scalability. The criteria system based on economy, environment and society factors in SSS, and the order allocation model with maximum purchasing value, minimum total cost and carbon dioxide emissions can be implemented in other practical applications. Therefore, the SSS-OA framework (Fig 1) is suitable for different types of manufacturing enterprises committed to sustainable development, such as household appliances, furniture production, electronic equipment, automobile industry, aviation industry, etc.

### 6.3 Limitations and future work

However, there are some limitations in this paper. Although we integrated the subjective method and objective method in the criteria weighting stage, the degree of differentiation in the subjective and objective dimensions is poor. In order to make up for this deficiency, we will strive to propose a comprehensive weight method that can fully show the discrimination degree between subjective and objective weights. Secondly, TODIM method fully considers the psychological factors of DMs to avoid risks, but the degree of risk avoidance of each individual is different in actual decision. We will consider applying the neural network method to the simulation of DMs’ psychological behavior. Artificial Intelligence is one of the main methods to solve decision-making problems. In the future, the integration of Artificial Intelligence technique and MCGDM method is a novel and perspective research direction.
